# Validation of biofilm formation on human skin wound models and demonstration of clinically translatable bacteria-specific volatile signatures

**DOI:** 10.1038/s41598-018-27504-z

**Published:** 2018-06-21

**Authors:** Mohammed Ashrafi, Lilyann Novak-Frazer, Matthew Bates, Mohamed Baguneid, Teresa Alonso-Rasgado, Guoqing Xia, Riina Rautemaa-Richardson, Ardeshir Bayat

**Affiliations:** 1 Plastic & Reconstructive Surgery Research, Division of Musculoskeletal & Dermatological Sciences, School of Biological Sciences, University of Manchester, Manchester, UK; 2 Manchester University NHS Foundation Trust, Wythenshawe Hospital, Manchester, UK; 3 Bioengineering Group, School of Materials, University of Manchester, Manchester, UK; 4 Manchester Academic Health Science Centre, Division of Infection, Immunity and Respiratory Medicine, School of Biological Sciences, Faculty of Biology, Medicine and Health, Manchester Academic Health Science Centre, The University of Manchester and Manchester University NHS Foundation Trust, Manchester, UK; 5 MCBA Consulting, Cardiff, Wales UK; 6 Division of Infection, Immunity and Respiratory Medicine, Faculty of Biology, Medicine and Health, Manchester Academic Health Science Centre, University of Manchester, Manchester, UK

## Abstract

Biofilms are major contributors to delayed wound healing and there is a need for clinically relevant experimental models to assess theranostics. Microorganisms release volatile organic compounds (VOCs) and the ability to identify these in infected cutaneous wounds could lead to efficient non-invasive diagnosis. The aims here were to develop and assess bacterial biofilm formation and identify their VOC profiles in an *in vitro* model and validate in human *ex vivo* incisional and excisional cutaneous wound models. Biofilm development was assessed using multiple microscopy techniques with biofilm-forming deficient controls and quantified using metabolic and biomass assays; and VOC production measured by gas chromatography-mass spectrometry. The production of most VOCs was affected by biofilm development and model used. Some VOCs were specific either for planktonic or biofilm growth. The relative abundance of some VOCs was significantly increased or decreased by biofilm growth phase (P < 0.05). Some *Staphylococcus aureus* and *Pseudomonas aeruginosa* VOCs correlated with biofilm metabolic activity and biomass (R ≤ −0.5; ≥0.5). We present for the first time bacterial biofilm formation in human *ex vivo* cutaneous wound models and their specific VOC profiles. These models provide a vehicle for human skin-relevant biofilm studies and VOC detection has potential clinical translatability in efficient non-invasive diagnosis of wound infection.

## Introduction

Biofilms are defined as complex microbial communities embedded in a protective self-produced biopolymer matrix, which provides protection against antimicrobial agents and host defence mechanisms^1^. Biofilms are a major contributor to delayed wound healing^2,3^ and there is an urgent need for clinically relevant biofilm experimental models to allow the development of wound infection theranostics. The porcine skin/wound substrate is the commonest *ex vivo* model used for biofilm experimentation^4^. Anatomically and physiologically, porcine skin is similar to human skin^5^, however it is not biologically or structurally identical. There are multiple methods used in the assessment of biofilm in experimental models^6^. Biofilms can be visualised and quantified using multiple microscopy techniques. Scanning electron microscopy (SEM) provides high resolution morphological and structural characterisation of the biofilm^7^. Epifluorescent microscopy can be used to visualise micro-colony formation and also quantify biofilm viability using fluorescent live/dead stains or selective probes that target bacteria specific gene sequences^8^. Other techniques include but are not limited to enumeration, colorimetric methods, metabolic and biomass assays^9^.

Current wound infection diagnosis involves clinical judgement in combination with microbiological analyses of wound swabs. Clinicians rely heavily on clinical wound characteristics for the diagnosis of infection^10^. These “classical” characteristics include oedema, erythema, warmth and purulence. However, there is uncertainty as to how accurate the presence of these characteristics, correlates with wound infection^11^. Additionally, these signs are not apparent until an infection is well-established. Laboratory-based techniques; both non- and culture based techniques, are time-consuming and culture over-estimates rapidly dividing non-fastidious bacteria and under-estimates more fastidious anaerobes^12^. Therefore, untargeted empirical antimicrobial treatment is common, causing delays in optimal wound management as well as risks for development of antimicrobial resistance.

Volatile organic compounds (VOCs) include a diverse group of carbon-based molecules (alcohols, isocyanates, ketones, aldehydes, hydrocarbons and sulphides) some of which are gaseous at ambient temperatures^13^. Increasing evidence demonstrates that VOCs are unique to various disease states and their early detection could represent a useful means of diagnosis^14–16^. Breath analyses of VOCs released by microorganisms is already being used to diagnose pulmonary infection^17^. VOC sampling has the advantage of being painless, non-invasive and reproducible. Early identification of VOCs in cutaneous wound infections could provide a non-invasive and effective method of diagnosis prior to the onset of gross malodour or obvious tissue reaction and damage.

**Figure 1:**
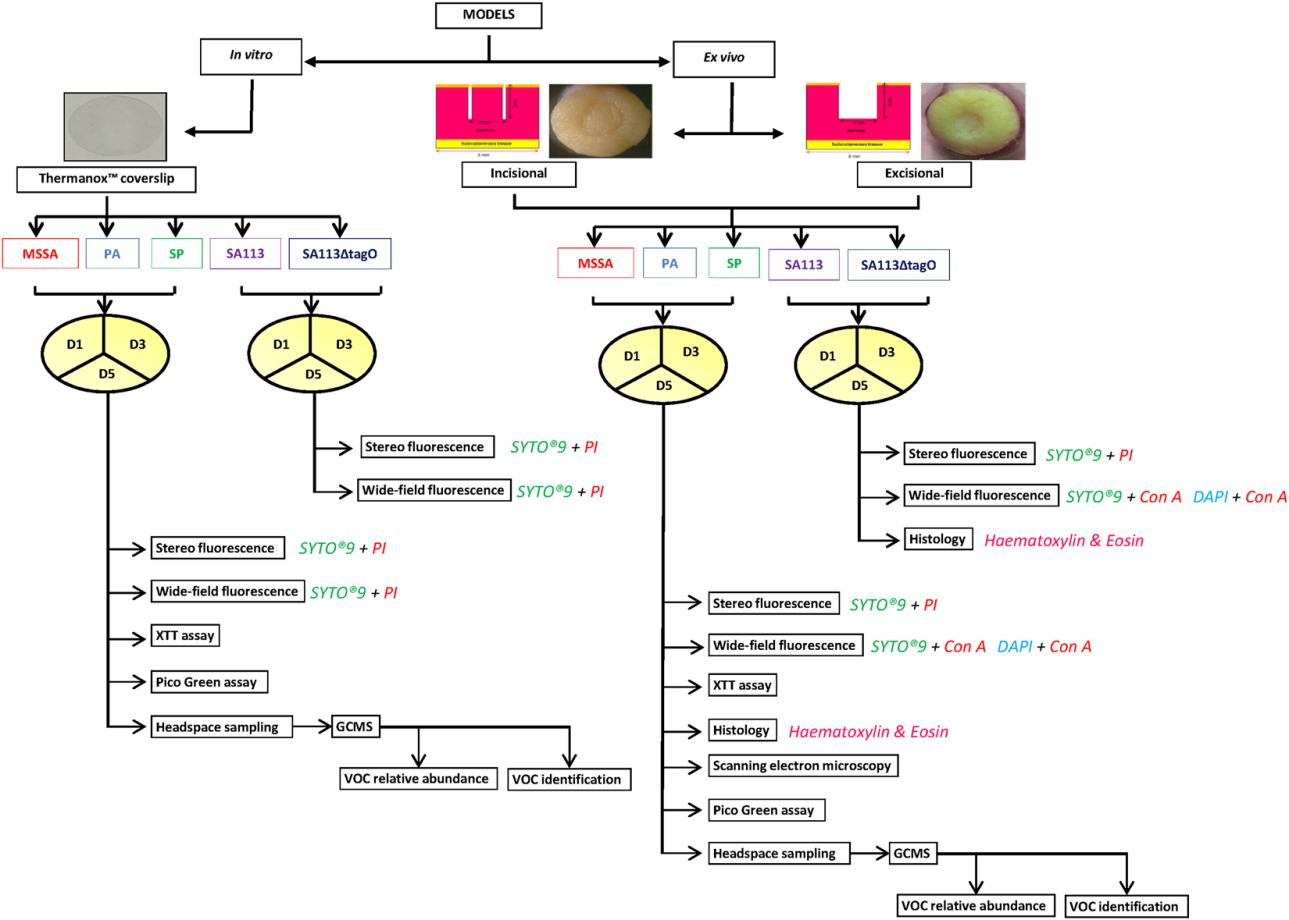
Study design. Biofilm formation of five bacterial species was evaluated in three models (one *in vitro* and two *ex vivo*) on three days (D1, 3 and 5) using multiple techniques and VOCs were identified on four days (D0, 1, 3 and 5). MSSA, methicillin sensitive *Staphylococcus aureus* ATCC 29213; PA, *Pseudomonas aeruginosa*; SP, *Streptococcus pyogenes*; SA113, Staphylococcus aureus wild type strain; SA113ΔtagO, biofilm-forming deficient mutant derived from SA113; VOCs, volatile organic compounds; PI, propidium iodide; Con-A, Concanavalin A; GCMS, gas chromatography mass spectroscopy.

Human *ex vivo* cutaneous wound models have been optimised for wound healing^18^. However, no previous studies have utilised human *ex vivo* incisional and excisional cutaneous wound models for bacterial biofilm formation, providing relevance to surgical and open wound cutaneous defects, respectively. Nor has VOC detection been utilised in the diagnosis of cutaneous wound infections. Therefore, the aims here were to develop and assess bacterial biofilm formation and identify their unique VOC profiles in an *in vitro* model and validate these using human *ex vivo* incisional and excisional cutaneous wound models. Biofilms were grown on plastic coverslips, incisional and excisional human cutaneous wound tissue explants in broth medium at 37 °C for 1, 3 and 5 days. Six different methods were used to evaluate biofilm formation. Histological assessment, stereo-fluorescence microscopy, wide-field fluorescence microscopy and SEM were used to visualise biofilm structure. XTT cell proliferation assay was used to determine biofilm metabolism and the amount of double stranded DNA was used to reflect biofilm biomass (Fig. 1). VOCs were identified using gas chromatography-mass spectrometry (GCMS). All experiments were done twice in triplicate.

## Results

### Species-dependent variation in biofilm formation in an *in vitro* cell culture model

**Figure 2:**
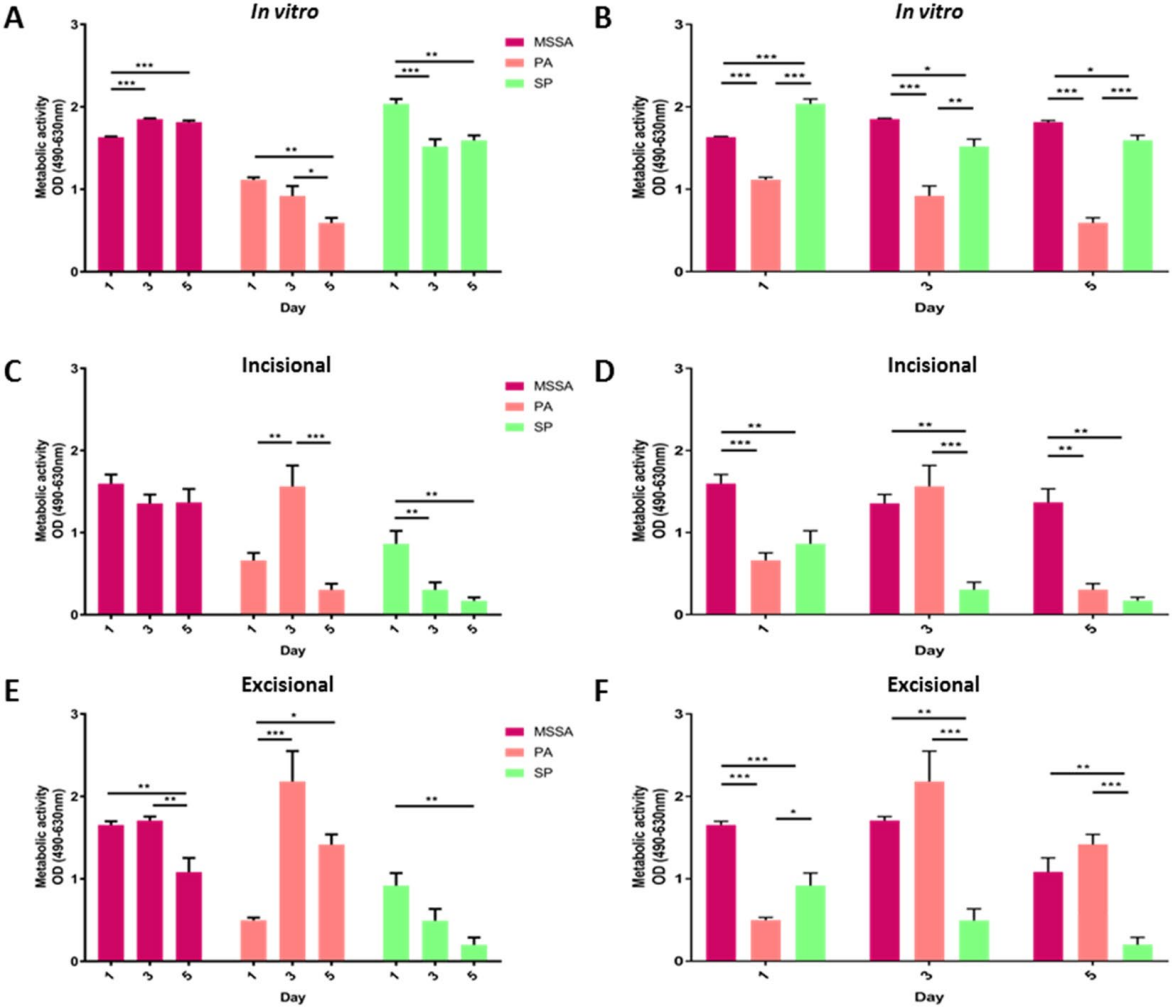
Metabolic activity of biofilms. Intra-strain comparisons of metabolic activity of (**A)** *in vitro*, (**C)** *ex vivo* incisional and (**E)** *ex vivo* excisional wound bacterial biofilms following XTT reduction assay. Inter-strain comparisons of metabolic activity of (**B)** *in vitro*, (**D)** *ex vivo* incisional and (**F)** *ex vivo* excisional wound bacterial biofilms following XTT reduction assay. Mean ± standard error of the mean (n = 6), *P < 0.05, **P < 0.01, ***P < 0.001, as determined by one-way analysis of variances with accompanying Tukey post hoc analyses.

**Figure 3:**
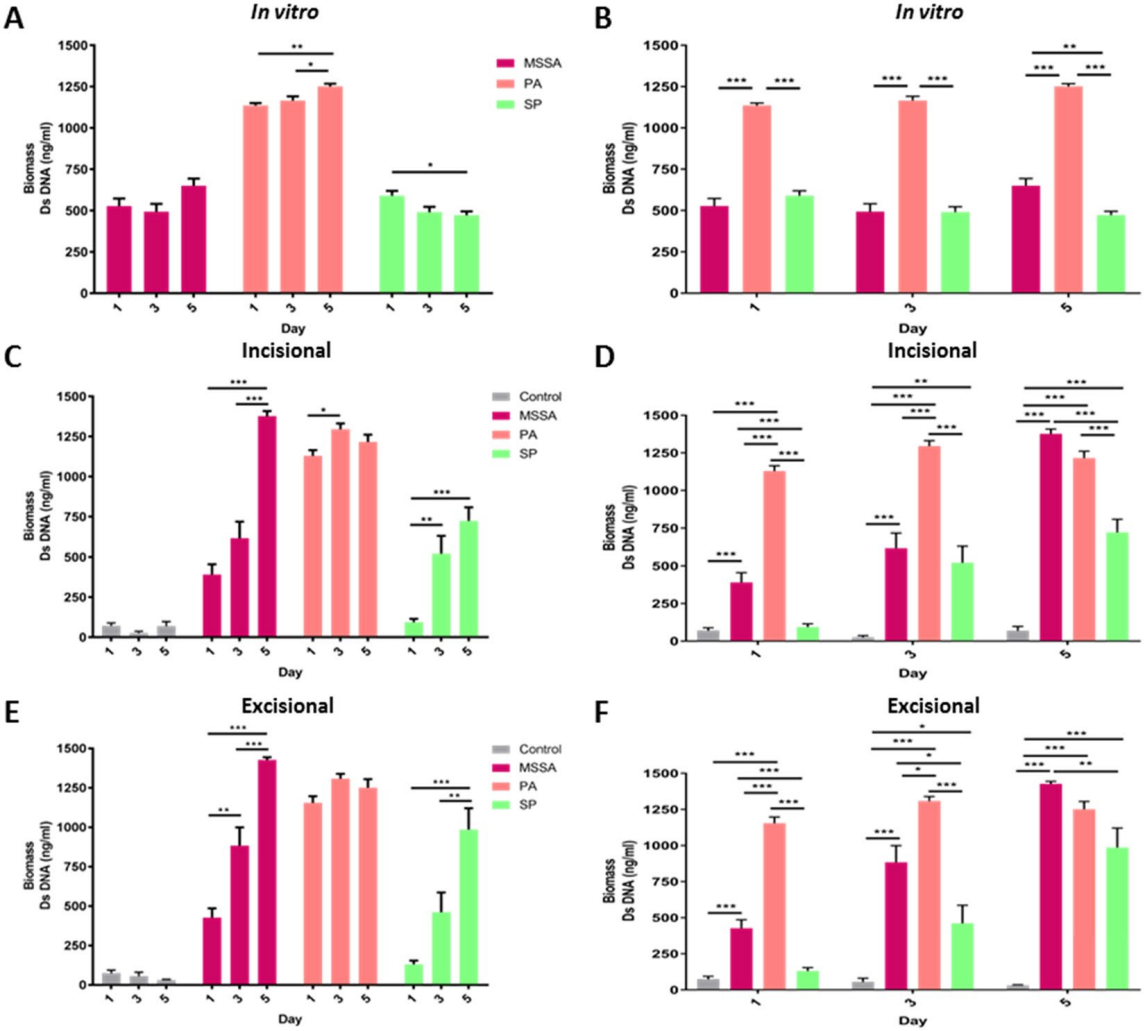
Biomass of biofilms. Intra-strain comparisons of biomass of (**A)** *in vitro*, (**C)** *ex vivo* incisional and (**E)** *ex vivo* excisional wound bacterial biofilms following Quant-iT PicoGreen dsDNA reagent assay. Inter-strain comparisons of biomass of (**B)** *in vitro*, (**D)** *ex vivo* incisional and (**F)** *ex vivo* excisional wound bacterial biofilms following Quant-iT PicoGreen dsDNA reagent assay. Mean ± standard error of the mean (n = 6), *P < 0.05, **P < 0.01, ***P < 0.001, as determined by one-way analysis of variances with accompanying Tukey post hoc analyses.

Biofilm development *in vitro* was monitored by viability staining, metabolic and biomass assays and confirmed to be present when evidenced by presence of an extracellular matrix (ECM), observed by microscopy as dispersed red material surrounding bacterial cells. Methicillin-sensitive *Staphylococcus aureus* ATCC 29213 (MSSA) showed early signs of biofilm formation, with the presence of viable bacteria interspersed between dead cells and limited ECM on day 1 (Fig. S1A,B). MSSA biofilms matured by day 5, evident as areas of heavily concentrated, mainly viable bacteria surrounded by ECM but also containing a mixture of dead cells and extracellular DNA stained red. This development and maturity is clear in contrast to the biofilm-deficient *Staphylococcus aureus* mutant (SA113ΔtagO) derived from the wild type methicillin-sensitive *Staphylococcus aureus* strain (SA113)^19^, where there is presence of viable bacteria (stained green) but lacking ECM. Relative to MSSA, mature *Pseudomonas aeruginosa* (PA) biofilm formation was evident by day 3, with a macroscopically detectable layer of slime, containing predominantly dead, but also interspersed with some viable cells by day 5. *Streptococcus pyogenes* (SP) showed delayed biofilm formation although by day 3, separate viable and dead bacterial cells surrounded by ECM were present. By day 5, viable nodules containing both live and dead cells were observed, suggestive of foci for dissemination and another hallmark of maturing biofilms (Fig. S1A,B). In addition to varying in biofilm formation and maturation microscopically, all three bacterial species showed temporal differences in metabolic activity (Fig. 2) and biomass (Fig. 3) with significant intra- and inter-strain differences (P < 0.05).

### Biofilm formation, viability, maturation and incorporation in *ex vivo* wound models

**Figure 4:**
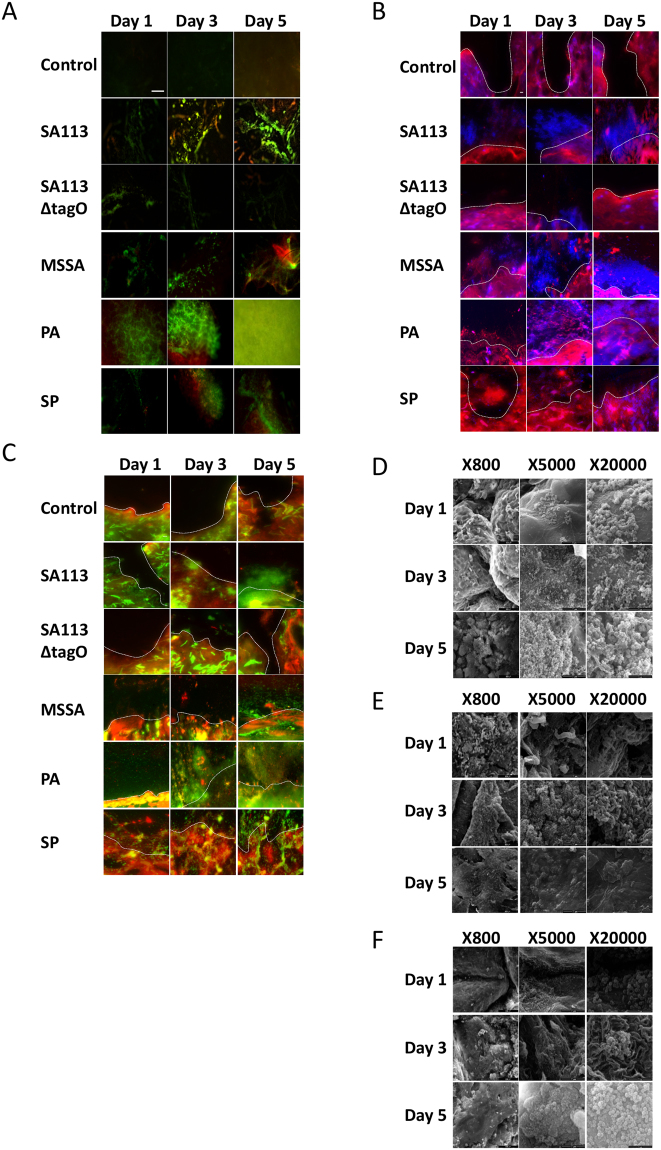
*Ex vivo* human incisional cutaneous wound model of biofilm formation. Representative images of control, SA113, SA113ΔtagO, MSSA, PA and SP biofilms after 1, 3 and 5 days incubation. **(A)** Stereo-fluorescence aerial microscopy with LIVE/DEAD® (SYTO® 9 and PI) staining. Viable cells are stained green and non-viable cells are stained red. Scale bar: 250 µm. Wide-field fluorescence microscopy of cross-sections stained with DAPI + Con-A **(B)** and SYTO® 9 + Con-A **(C)**. Viable bacterial cells are stained blue (DAPI) or green (SYTO® 9) and ECM is stained red (Con-A). White dashed line **(B,C)** indicates biofilm/wound tissue boundary. Scale bar: 10 µm. Scanning electron microscopy (SEM) images of MSSA **(D)**, PA **(E)** and SP **(F)** biofilms. SA113, *Staphylococcus aureus* wild type strain; SA113ΔtagO, biofilm-forming deficient mutant derived from SA113; MSSA, methicillin sensitive *Staphylococcus aureus* ATCC 29213; PA, *Pseudomonas aeruginosa*; SP, *Streptococcus pyogenes*; PI, propidium iodide; DAPI, 4’,6-diamidino-2-phenylindole; Con-A, Concanavalin A.

**Figure 5:**
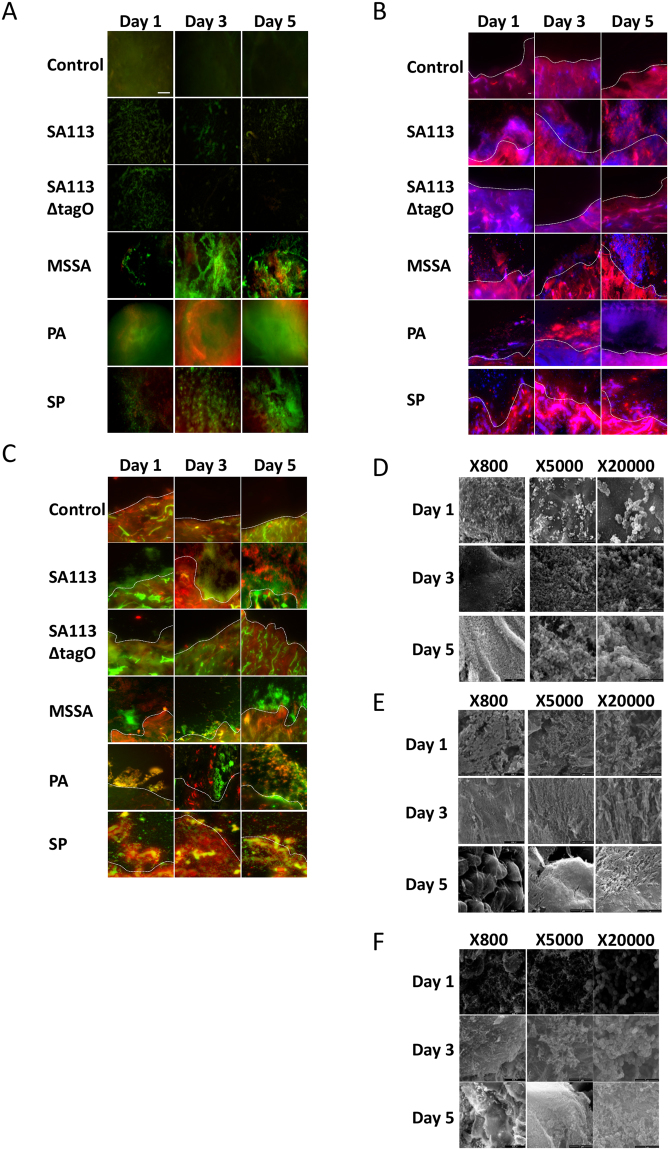
*Ex vivo* human excisional cutaneous wound model of biofilm formation. Representative images of control, SA113, SA113ΔtagO, MSSA, PA and SP biofilms after 1, 3 and 5 days incubation. **(A)** Stereo-fluorescence aerial microscopy with LIVE/DEAD® (SYTO® 9 and PI) staining. Viable cells are stained green and non-viable cells are stained red. Scale bar: 250 µm. Wide-field fluorescence microscopy of cross-sections stained with DAPI + Con-A **(B)** and SYTO® 9 + Con-A **(C)**. Viable bacterial cells are stained blue (DAPI) or green (SYTO® 9) and ECM is stained red (Con-A). White dashed line **(B,C)** indicates biofilm/wound tissue boundary. Scale bar: 10 µm. Scanning electron microscopy (SEM) images of MSSA **(D)**, PA **(E)** and SP **(F)** biofilms. SA113, *Staphylococcus aureus* wild type strain; SA113ΔtagO, biofilm-forming deficient mutant derived from SA113; MSSA, methicillin sensitive *Staphylococcus aureus* ATCC 29213; PA, *Pseudomonas aeruginosa*; SP, *Streptococcus pyogenes*; PI, propidium iodide; DAPI, 4′,6-diamidino-2-phenylindole; Con-A, Concanavalin A.

There was no significant difference in explant viability over the 5 day period in both *ex vivo* models (P > 0.05; data not shown). In comparison to the development observed *in vitro*, biofilm formation in *ex vivo* models was initially delayed. However, by day 5, all the hallmarks of biofilm formation, development and maturation were observed, with MSSA and SP forming more prolific biofilms by day 5 in *ex vivo* models compared to *in vitro* (Figs 2–5A, S2 and S3).

Viability staining revealed adherent MSSA cells on day 1 and signs of early biofilm formation by Day 3, observed as viable bacterial colonies interspersed in limited ECM (Figs 4A and 5A). On day 5, the hallmarks of biofilm formation were more evident, with viable bacterial foci encased in thick ECM, particularly in comparison to biofilm-deficient mutant strain SA113ΔtagO (Fig. 5A). A combination of Concanavalin-A (Con-A) and 4′,6-diamidino-2-phenylindole (DAPI) staining, confirmed early colonisation followed by MSSA biofilm formation, with the presence of ECM surrounding bacterial cells by day 3 and thickening with time (Fig. 4B). By day 5, there was a prolific, thick biofilm present with viable, distinct bacteria (stained blue) encased in ECM, similar to the positive control SA113 biofilm. Staining with an alternative combination of Con-A and SYTO^®^ 9 (Figs 4C and 5C) and haematoxylin and eosin supported these findings, revealing pillar-like formations and MSSA biofilm formation within the wound (Figs S4 and 5). Scanning electron microscopy (SEM) further confirmed MSSA colonisation on day 1, bacteria interspersed within ECM on day 3 and multi-layered biofilm prolific with MSSA cells, both within the wound and the surrounding skin (Figs 4D and 5D). This was supported by decreasing metabolic activity, known to occur in mature biofilms and a significant increase in biomass (P < 0.05; Figs 2 and 3).

PA biofilms formed and proliferated faster than MSSA biofilms in both *in vitro* and *ex vivo* models based on viability staining (Figs 4A, 5A and S1A,B) and biomass formation (Fig. 3). Staining with Con-A in combination with DAPI or SYTO^®^ 9, revealed both viable (stained blue or green, respectively) and dead (stained red) PA cells embedded in extensive ECM by day 3 (Figs 4B,C and 5B,C). Mature PA biofilms exhibited a macroscopically detectable layer of slime which masked visibility of individual cells in fluorescently stained materials (Figs 4 and 5) but which were clearly visible in haematoxylin and eosin stained sections where ECM appeared blue and host tissue stained pink (Figs S4 and 5). SEM confirmed the presence of rod-shaped PA cells on day 3, interconnected by a relatively thick ECM in comparison to MSSA (Figs 4D,E and 5D,E), particularly in the excisional model (Fig. 5E). By day 5, it was difficult to visualise individual PA cells within the slime surrounding the explant surface (Figs 4E and 5E), although metabolic assays confirmed PA cell proliferation during biofilm development and at maturity (Fig. 2).

Adherence of SP to the substratum was evident on day 1 (Fig. 4A), particularly in the excisional wound model (Fig. 5A). Even though, *in vitro* SP biofilms were easily distinguishable on day 3 (Fig. S1A,B), presence of SP biofilms in the *ex vivo* models, including production of diffuse-staining ECM, was evident only by day 3 (Figs 4A and 5A). Clearly SP biofilm formation was delayed compared to MSSA and PA (Figs 4A–C and 5A–C), although most SP bacteria encased in ECM remained viable even by day 5 as shown by the green staining (Figs 4A,C and 5A,C). Thickened ECM was clearly visible by SEM from day 3 (Figs 4F and 5F) as was the presence of SP cells, not only on the surface of the wound but also within the tissue (Figs S4, 5). Moreover, SEM showed clearly the proliferation of SP biofilm from day 1 to 5 (Figs 4F and 5F) in keeping with increasing biomass (Fig. 3).

### VOCs are specific to bacterial species

**Figure 6:**
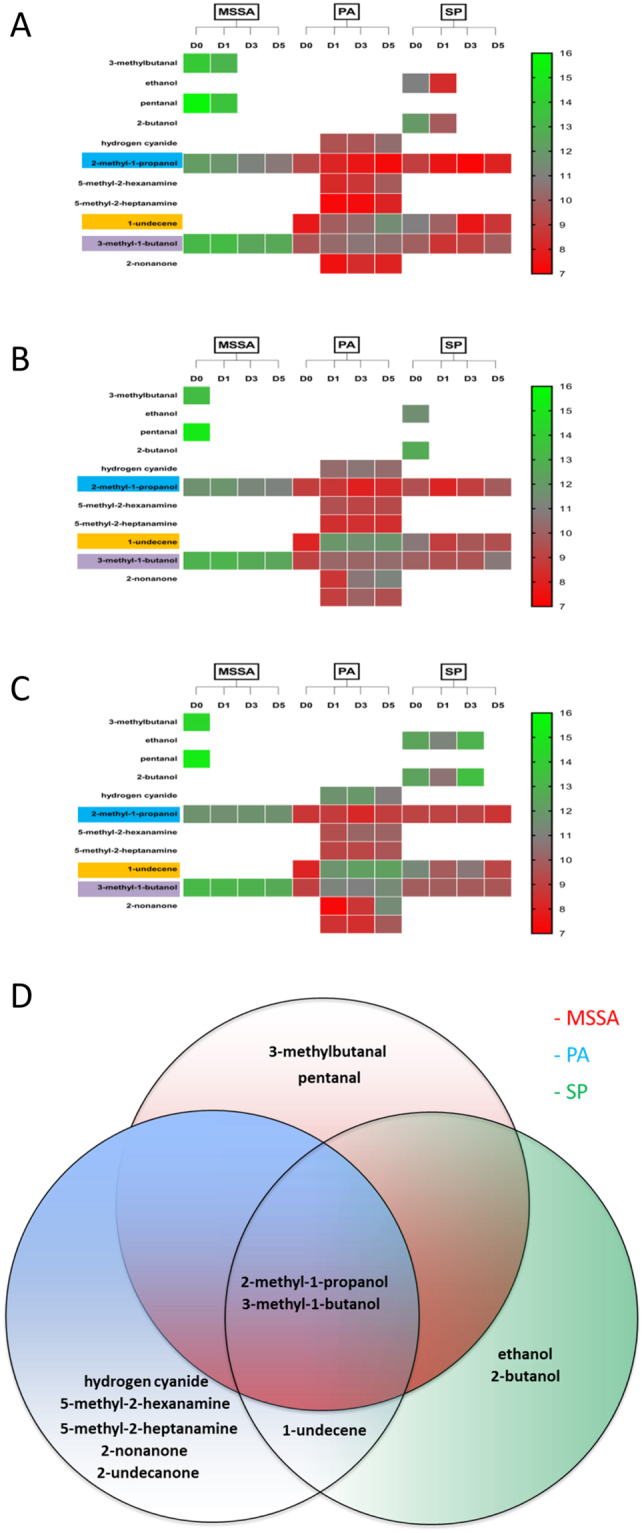
Heat map and Venn diagram representing the distribution of VOCs in different wound models. Heat map outlining the presence and relative abundance (chromatographic peak area) of a VOC based on bacterial species and time points in the *in vitro* **(A)**, *ex vivo* human cutaneous incisional **(B)** and excisional wound **(C)** models. **(D)** Venn diagram identifying VOCs unique to and shared between MSSA, PA and SP. MSSA, methicillin sensitive *Staphylococcus aureus* ATCC 29213; PA, *Pseudomonas aeruginosa*; SP, *Streptococcus pyogenes*; VOCs, volatile organic compounds.

VOC sampling of the biofilms cultured on human incisional and excisional cutaneous wound substrates were undertaken with relevance to surgical and open wound cutaneous defects *in vivo*. Six samples for each biofilm (MSSA, PA and SP) or equivalent non-infected controls (sterile Mueller Hinton Broth and Brain Heart Infusion Broth) were processed and analysed by GCMS at four time points (days 0, 1, 3 and 5) for *in vitro* and *ex vivo* incisional/excisional wound models culminating in a total of 360 samples. Twelve peaks unique to bacterial samples were identified (Table S1) after subtracting control peaks (Fig. S6). 3-methylbutanal and pentanal were unique to MSSA; hydrogen cyanide, 5-methyl-2-hexanamine, 5-methyl-2-heptanamine, 2-nonanone and 2-undecanone (in *ex vivo* human cutaneous wound models only) were unique to PA; and ethanol and 2-butanol were unique to SP (Fig. 6) suggesting these VOCs have the potential to differentiate between these species. In addition, 1-undecene was identified in PA and SP (highlighted in orange), and 2-methyl-1-propanol (highlighted in blue) and 3-methyl-1-butanol (highlighted in purple) were identified in all 3 species (Fig. 6). 2-methyl-1-propanol and 3-methyl-1-butanol were identified at all time points in all three bacteria grown either planktonically or as a biofilm. Notably, the relative abundance of these two compounds was significantly higher in all MSSA samples (P < 0.05) suggesting the relative abundance of some VOCs may allow species differentiation (Figs S7–9).

### Some VOCs are biofilm and model-specific

**Figure 7:**
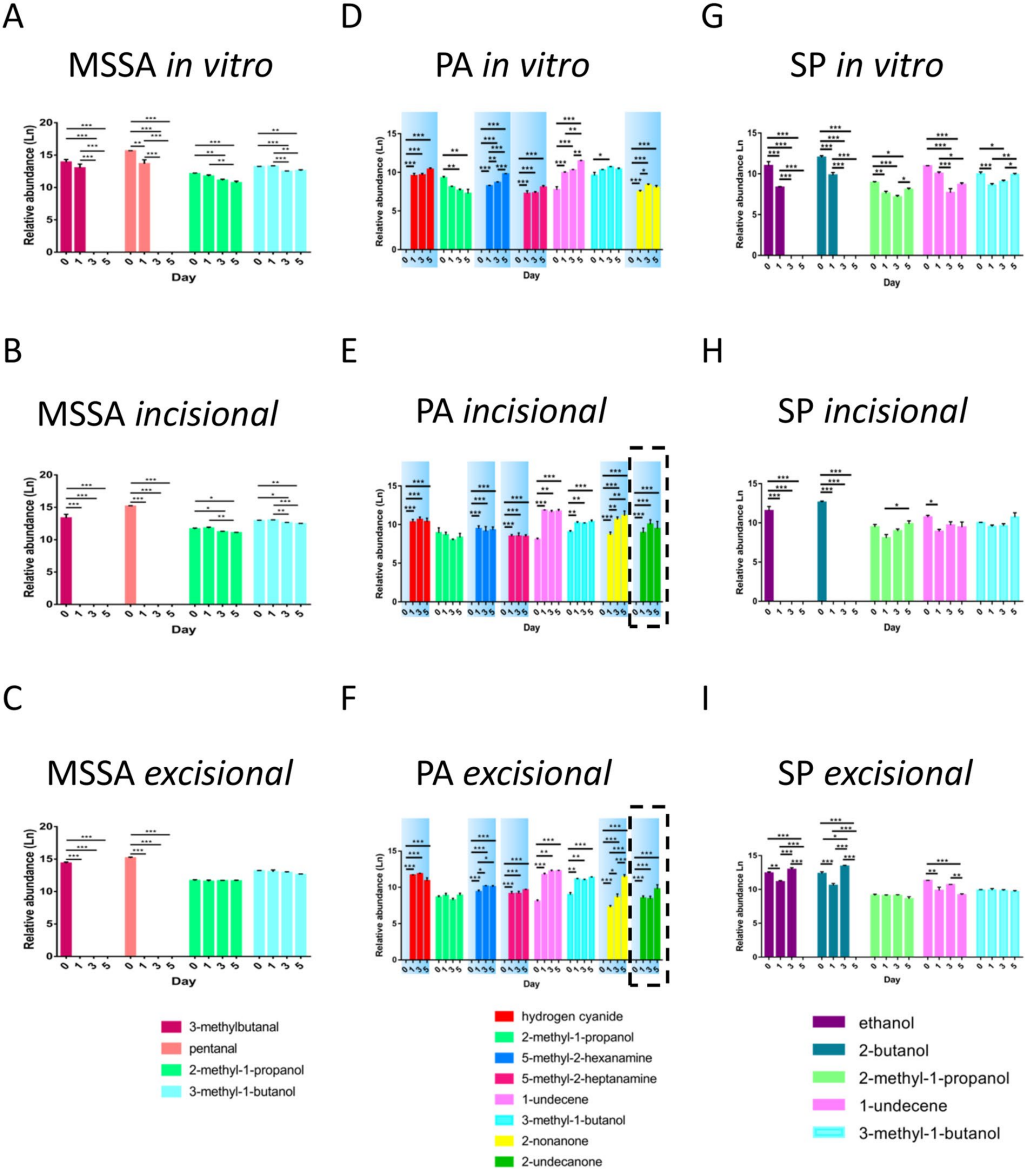
Identification of distinct VOCs and relative abundance of compounds in different wound models. Identification and relative abundance (chromatographic peak area) of VOCs in MSSA **(A–C)**, PA **(D–F)** and SP **(G**–**I)** grown in different biofilm models and monitored on days 0, 1, 3 and 5. Relative abundance of VOCs identified in *in vitro* **(A,D,G)**, *ex vivo* incisional **(B,E,H)** and excisional **(C,F,I)** wound models. Blue shading highlights VOCs specific to biofilms; dashed areas represents VOCs specific to compounds produced in the *ex vivo* models. Mean ± standard error of the mean (n = 6), *P < 0.05, **P < 0.01, ***P < 0.001, as determined by independent samples t-test. MSSA, methicillin sensitive *Staphylococcus aureus* ATCC 29213; PA, *Pseudomonas aeruginosa*; SP, *Streptococcus pyogenes*; VOCs, volatile organic compounds.

**Figure 8:**
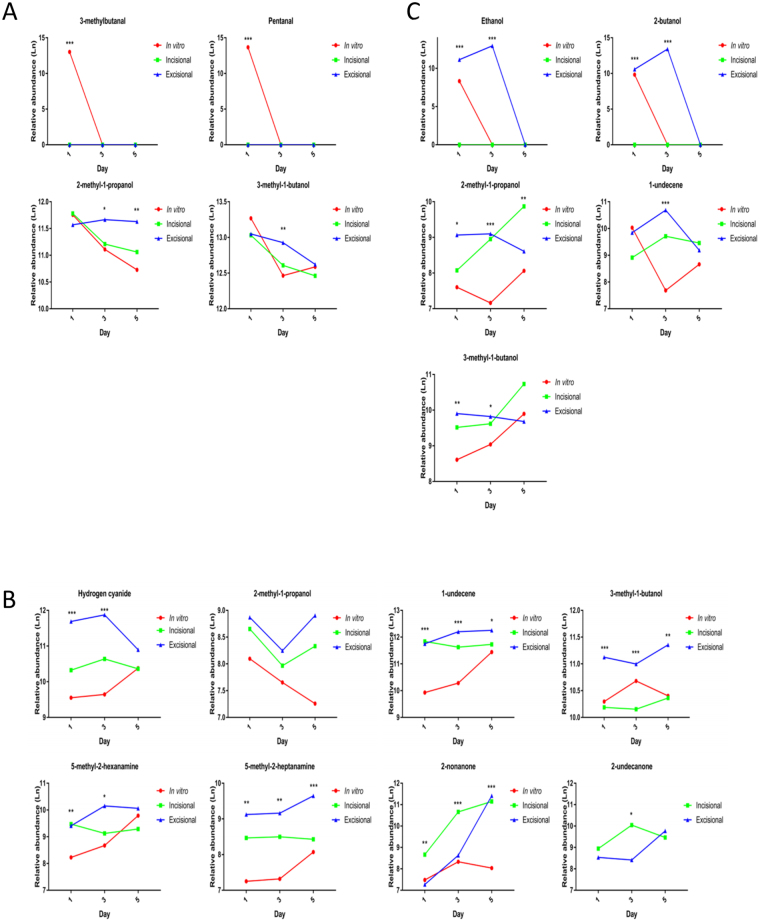
Comparison of VOCs and relative abundance of compounds produced at different time points in biofilm models. Presence and relative abundance of VOCs identified in MSSA **(A)**, PA **(B)** and SP **(C)** biofilms grown in *in vitro* and *ex vivo* models and monitored on days 0, 1, 3 and 5. Mean (n = 6), *P < 0.05, **P < 0.01, ***P < 0.001, as determined by one way-analysis of variance. MSSA, methicillin sensitive *Staphylococcus aureus* ATCC 29213; PA, *Pseudomonas aeruginosa*; SP, *Streptococcus pyogenes*; VOCs, volatile organic compounds.

In addition to bacteria-specific VOCs, their presence and abundance were influenced by biofilm growth phase (P < 0.05) and wound models (i.e. *in vitro* versus *ex vivo*) (P < 0.05) (Figs 7 and 8; Tables S2–4). Hydrogen cyanide, 5-methyl-2-hexanamine, 5-methyl-2-heptanamine, 2-nonanone and 2-undecanone were specific to PA biofilms and not identified in the corresponding planktonic bacterial phase (Fig. 7). Moreover, 2-undecanone was only detected in the two *ex vivo* PA cutaneous models (Fig. 7E,F), suggesting the potential for monitoring PA infections *in vivo* and highlighting the importance of selecting clinically relevant experimental models. In contrast, MSSA and SP did not produce any biofilm-specific VOCs (i.e. 3-methylbutanal, pentanal and ethanol, 2-butanol, were produced in planktonic cultures and biofilms, respectively, see Fig. 7).

1-undecene was identified in both planktonic and biofilms of PA and SP (Fig. 7D–I) but not in any MSSA samples (Figs 6 and 7A–C). Moreover, the relative abundance of 1-undecene was reciprocal in PA and SP biofilms and allowed differentiation during development (P < 0.05) (Fig. S9). The trend of hydrogen cyanide production in PA biofilms (Fig. 8) correlates with PA biomass (Fig. 3); and significantly increased production of 2-nonanone (P < 0.05), almost exclusively in established *ex vivo* PA wound biofilms (Fig. 7), may be correlated with ECM accumulation observed microscopically (see Figs 4E and 5E). These may monitor PA ECM production, which is associated with maturing biofilms.

### MSSA and PA biofilm production of specific VOCs is correlated with metabolic activity and biomass

MSSA metabolic activity correlated with 2-methyl-1-propanol (R = 0.5) and 3-methyl-1-butanol (R = 0.5) across the two cutaneous wound models. PA metabolic activity correlated with hydrogen cyanide (R = 0.5), 2-methyl-1-propanol (R = −0.5) and 5-methyl-2-heptanamine (R ≥ 0.5) across the two cutaneous wound models and with 3-methyl-1-butanol (R ≤ −0.5) across all three models (Fig. S10). MSSA biomass correlated with 3-methyl-1-butanol (R = −1) across the two cutaneous wound models. PA biomass correlated with 3-methyl-1-butanol (R = −0.5) across the two cutaneous wound models and with hydrogen cyanide (R ≥ 0.5), 2-methyl-1-propanol (R ≤ −0.5) and 5-methyl-2-heptanamine (R ≥ 0.5) and 2-nonanone (R = 0.5) across all three models (Fig. S11).

## Discussion

We have successfully established the formation, development and maturation of common cutaneous wound pathogenic bacterial biofilms on human *ex vivo* incisional and excisional cutaneous wound models. To our knowledge, this is the first report of bacterial biofilm formation on human *ex vivo* wound models, with a previous study utilising human skin as a substrate for *Candida* biofilm formation^20^. The porcine *ex vivo* skin/wound model is most commonly utilised for experimental biofilm formation^4,21–24^ as it is similar to human skin both anatomically and physiologically^5^. However, porcine skin is not identical to human skin and therefore it was necessary to develop skin-identical models to allow ease of clinical translatability of experimental findings.

Imaging of biofilms on human cutaneous explants using stereo-fluorescence microscopy requiring minimal tissue preparation showed marked differences in staining intensity between control and inoculated samples across the whole surface of the explant. The advantage of using stereo-fluorescence microscopy was the ability to image biofilms cultured on an opaque substrate, a requirement for imaging whole human and animal *ex vivo* biofilm models. However, due to magnification limitations, it was not possible to visualise individual bacteria within the biofilm. Thus, although wide-field fluorescence microscopy requires significant sample manipulation, with the possibility of biofilm disruption, it was necessary to visualise individual bacteria within the biofilms, which we confirmed for all three bacterial species in both cutaneous wound models. MSSA and PA biofilms generally formed more rapidly and matured more quickly than SP. This may be related to pathogenicity of the strains or the inherently faster growth rates of MSSA and PA generally. Haematoxylin and eosin staining confirmed biofilm incorporation into the cutaneous tissue during the latter time points and also confirmed MSSA and PA biofilm formation were more prolific compared to SP. Fluorescent staining allowed determination of bacterial viability, production of ECM and microscopic visualisation of the slime that was seen macroscopically which is indicative of the biofilm ECM^25^. SEM provided optimum visualisation of biofilm development and maturation for all three bacterial strains in both human cutaneous wound models because individual bacteria were clearly defined as well as the interconnecting ECM, characteristic of biofilms^26^. Visualisation of biofilm development, was confirmed through quantification of the biomass using PicoGreen assay^27^, which showed a temporal increase in biomass for all species.

We identified from GCMS analysis 12 chromatographic peaks associated with biofilm growth and development, some of which were shared among or unique to MSSA, PA and SP and some of which are newly described. The 12 peaks identified are solely as a result of the bacteria since chromatographic peaks present in both infected and non-infected samples were eliminated from further analysis as these peaks could possibly be attributed to the substrate upon which the biofilm was formed. 2-methyl-1-propanol, which has previously been found in a non-cutaneous wound setting MSSA culture^28^, was released in planktonic and biofilm phases of all 3 bacteria investigated, although its relative abundance was significantly higher in MSSA compared to the other species. The abundance of 2-methyl-1-propanol decreased significantly as MSSA biofilms developed and could potentially be used to differentiate MSSA biofilm maturity. In a clinical study, a propanol derivative, among other VOCs, allowed differentiation between chronic wounds and normal skin within individuals, possibly related to the variances in bacterial colonisation^16^. 3-methyl-1-butanol was also present in all species, stages of biofilm development and in all three wound models, however, significant differences in relative abundance between MSSA and the other two species allowed differentiation between species. Butanol and its derivatives, including 3-methyl-1-butanol, have been identified previously in PA, *E. coli*, MSSA and *Staphylococcus epidermidis*^29–32^. 1-undecene, an alkene previously shown to be produced by PA^28^, was present at all time points in both PA and SP biofilms in all three wound models. 1-undecene is likely a degradation product of fatty acids^33^. The relative abundance of 1-undecene increased significantly over time in PA, particularly in *ex vivo* wound models, but decreased significantly over time in SP biofilms suggesting the potential for using VOCs and their relative abundance to not only identify bacterial species but also to monitor biofilm development.

3-methylbutanal and pentanal were uniquely identified in the planktonic or very early biofilm phases of MSSA in all three models. This could potentially allow differentiation between planktonic/early biofilm phases from well-developed MSSA biofilms. 3-methylbutanal has been corroborated previously by Filipiak *et al*. who identified it was distinctive for MSSA over a similar time frame^28^. Aldehydes, such as pentanal, are common bi-products of bacterial metabolism^34^ and it has been identified in exhaled air of healthy human volunteers, even though not specifically associated with MSSA or other microorganisms^35^. Although, the association between hydrogen cyanide and PA is well documented in lung infections^36^, this is the first documentation of its production in a wound setting. 5-methyl-2-hexanamine and 5-methyl-2-heptanamine were found in PA biofilms at all time points, irrespective of biofilm development and wound model. Amines are nitrogen containing VOCs and have been found previously to be associated with MSSA, PA, *Enterococcus faecalis*, *Streptococcus pneumoniae*, *Escherichia coli* and *Klebsiella pneumoniae*^34^, however, this is the first documentation of amine production by PA in a cutaneous wound model. We confirmed the presence of 2-nonanone in PA, and showed that this VOC was produced in greater relative quantities in an *ex vivo* biofilm setting, suggesting its potential as an indicator VOC. Identification of 2-undecanone specific to our *ex vivo* PA models confirms that this compound is associated with PA biofilm infection in a wound setting and demonstrates the importance of substrate selection on VOC production. Moreover, our demonstration of the relationship between relative abundance of hydrogen cyanide and 2-nonanone coincident with ECM production, suggests their importance during PA biofilm development in a wound setting. Ethanol and 2-butanol were identified in SP cultured on both incisional and excisional cutaneous wound explants, where their production was enhanced, as well as in the *in vitro* model. Interestingly, ethanol and 2-butanol production, which have previously been observed in MSSA^28,37^ and PA^31^, were not observed in our MSSA and PA biofilm wound settings which may further affirm the importance of substrate selection on VOC production.

VOC production is a bi-product of bacterial metabolism^33^ and we identified a relationship between VOC relative abundance and metabolic activity which was consistent across all three models or across the *ex vivo* models. DNA is a major component of the biofilm ECM^38,39^. We found significant increases in the biomass of both MSSA and SP in both *ex vivo* models over time. This trend was not seen in the *in vitro* model and the biofilm was more prolific in the tissue models, highlighting the importance of selecting appropriate models in biofilm studies. The associations we found are of possible interest as the use of VOCs could not only be used to identify causal organisms but their abundance could be extrapolated to show progression of biofilm development and maturity as an indicator of the severity of infection; however, further evaluation is necessary.

This study has a number of limitations. Only three bacterial strains were used as mono-cultures, although most chronic wounds exhibit as poly-microbial biofilms^2^ and VOC production may be influenced by presence of more than one species^40^. However, single pathogen wound infections do occur. Also, as this was an *ex vivo* study the medium selection and growth conditions may have had an impact on the VOCs. However, we were able to detect the same VOCs for different strains grown in different nutrient broths, suggesting these factors may not be that significant when the appropriate substrate is used. An internal standard to account for possible GCMS variations over time was not used; instead we identified a common peak to all (infected and non-infected) samples and normalised all peaks to this common VOC. An internal standard was not used due to concerns of possible interactions with VOCs of interest. Finally, the identification of the compounds using the mass spectral library must be interpreted with caution as they have not been confirmed by analytical standards.

In conclusion, we successfully cultured and described the characteristics of three pathogenic bacterial biofilms commonly found in wounds in two human *ex vivo* cutaneous wound organ culture models. We showed that these models may have a role in experimental biofilm studies with the potential for clinical translatability. Additionally, we identified VOCs that allow bacterial species differentiation and assess biofilm development. These findings have potential clinical applicability for the efficient and non-invasive diagnosis of cutaneous wound infection and in order to confirm these possibilities, clinical studies using novel headspace accumulation chambers are underway.

## Methods

### Bacterial strains

Bacterial isolates were American Type Culture Collection reference strains obtained directly from LGC standards (Teddington, UK). The bacterial strains used in this study were *Staphylococcus aureus* ATCC 29213 (MSSA) which is a methicillin-sensitive wound isolate; *Pseudomonas aeruginosa* ATCC 27316 (PA) which is a human wound isolate; and *Streptococcus pyogenes* ATCC 700294 (SP) which is an infected wound isolate. Additionally, wild type methicillin sensitive *Staphylococcus aureus* (SA113) and its biofilm-deficient *Staphylococcus aureus* (SA113ΔtagO) derivative were used as additional control strains^19^. All strain stocks were frozen and stored long term at −80 °C. All isolates were revived onto Columbia agar containing horse blood (BA, E&O Laboratories Ltd, Bonnybridge, Scotland) and incubated at 37 °C for 16–18 h prior to use.

### Apparatus setup

A 20 ml precision thread headspace vial (Fisher Scientific, Loughborough, UK) with a universal magnetic aluminium screw cap with PTFE/butyl penetrable septum (Fisher Scientific, Loughborough, UK) was chosen as the optimum apparatus for both biofilm formation and accumulation and containment of volatile organic compounds (VOCs) to allow headspace sampling and analysis by gas chromatography-mass spectrometry (GCMS). This apparatus was chosen as it provided a concentrated headspace volume to allow VOC detection and was efficient for GCMS auto sampling.

### *Ex vivo* cutaneous incisional and excisional wound organ culture models

Full thickness cutaneous tissue was obtained from human patients (n = 16; 15 female; mean age 44.9 years) undergoing elective surgery (10 abdominoplasty; 5 breast reduction; 1 brachioplasty) with appropriate ethical approval (16/NW/0736). The skin underwent intra-operative disinfection with aqueous chlorhexidine: tissue was trimmed of excess adipose tissue and disinfected with 2% chlorhexidine gluconate and 70% isopropyl alcohol (ChloraPrep™ Sepp™ applicator, BD, Basingstoke, UK) by rubbing the skin surface gently for 30 s. A sample of disinfected tissue was cultured both aerobically and anaerobically for 24 h onto BA to confirm elimination of normal skin flora. Eight mm circular biopsies were taken from the main tissue sample using disposable biopsy punches (Integra™ Miltex^®^, York, PA, USA). In the centre of these circular skin explants, a 3 mm diameter circular incisional or excisional artificial wound was created to a depth of 2 mm by a further punch biopsy. Explants were inserted into sterile 24-well Corning® Costar® cell culture plates (Sigma-Aldrich, Poole, UK), with each well containing 0.5 ml serum-free Williams E culture medium (ThermoFisher Scientific™, Basingstoke, UK) supplemented with 1% non-essential amino acid solution, 10 µl/ml insulin, 10 ng/ml hydrocortisone and 2mmol L-glutamine. Explants were maintained at 37 °C in a stationary carbon dioxide (5%) incubator and were cultured for a minimum of 24 h with daily medium change prior to use.

### Biofilm formation in an *in vitro* cell culture model

Frozen stocks of MSSA, PA, SP, SA113 and SA113ΔtagO were streaked onto BA to isolate single colonies and incubated in a stationary incubator at 37 °C for 16–18 h. To generate a supply of cells in broth medium a 50 ml centrifuge tube containing 25 ml Mueller Hinton Broth was inoculated with a single colony of MSSA, PA, SA113 or SA113ΔtagO and incubated at 37 °C in a shaking incubator at 250 rpm overnight. A 50 ml conical centrifuge tube containing 25 ml Brain Heart Infusion Broth was inoculated with a single colony of SP and incubated in a stationary incubator at 37 °C overnight. Inoculated broth was then diluted to 0.9–1.1 OD with appropriate sterile broth medium the following day. A range of 0.9–1.1 OD was set across all three species to equate to bacterial count greater than 10^8^ CFU/ml. This technique allowed an efficient method for bacterial count determination prior to inoculation. This equated to a bacterial count of ~10^9^ CFU/ml (range: 5 × 10^8^–4 × 10^9^ CFU/ml) selected for preparation of biofilms.

A 13 mm diameter Nunc™ Thermanox™ Coverslip (Thermo Scientific, Hampshire, UK) was used as the inert substrate for biofilm formation. This coverslip is 0.2 mm thick and is treated on one side with a proprietary polyester coating modified to be hydrophilic to allow increased cell adherence. A single coverslip was placed with the treated surface facing upwards into a sterile 24-well Corning® Costar® cell culture plate. 1 ml of MSSA, PA, SP SA113 or SA113ΔtagO bacterial suspension at 0.9–1.1 OD was pipetted into the well submerging the plastic coverslip. The plates or vials were then incubated at 37 °C in a stationary carbon dioxide (5%) incubator for up to 1, 3 or 5 days. Appropriate controls inoculated with 1 ml sterile Mueller Hinton or Brain Heart Infusion broths were also prepared and incubated for up to 1, 3 and 5 days.

### Biofilm formation on *ex vivo* cutaneous wound organ culture models

A supply of bacterial cells in broth medium was generated as described above. Individual 8 mm incisional or excisional cutaneous wound explants were used as the substrate for biofilm formation. The explant was placed with the wounded surface facing upwards into a sterile 24-well Corning® Costar® cell culture plate. Bacterial suspensions of MSSA, PA or SP (1 ml at 0.9–1.1 OD) were pipetted into a well submerging a wound explant. The plates or vials were then incubated at 37 °C in a stationary carbon dioxide (5%) incubator for up to 1, 3 or 5 days. Appropriate controls (explants without inoculum) supplemented with 1 ml of sterile Mueller Hinton or Brain Heart Infusion broth were also prepared and incubated in the same manner. Samples of inoculated tissue were cultured aerobically for 24 h onto BA to confirm mono-microbial biofilm formation at 1, 3 and 5 days.

### Stereo-fluorescence microscopy

Inoculated and control coverslips, incisional and excisional wound tissue explants were stained at days 1, 3 and 5 with FilmTracer™ LIVE/DEAD^®^ Biofilm Viability Kit (Invitrogen Molecular Probes^®^, NY, USA) to visualise biofilm formation and assess cell viability. When used alone, SYTO^®^ 9 stain labels all bacteria in the biofilm. In contrast, propidium iodide (PI) penetrates bacteria with damaged cell membranes and binds to double stranded DNA by intercalating between base pairs, causing a reduction in SYTO^®^ 9 staining when both dyes are present in the cell. Therefore, when present in an appropriate mixture, bacteria with intact cell membranes stain fluorescent green, whereas bacteria with damaged membranes stain fluorescent red. Fluorescent stain was prepared by adding 3 µl SYTO^®^ 9 and 3 µl PI in 1 ml sterilised distilled water. Each coverslip was washed in sterilised, distilled water before 200 µl staining solution was added gently to the biofilm and incubated for 20 min at room temperature in the dark. Coverslips or explants were then rinsed gently with distilled water to remove excess stain and images were collected and captured using a Leica MZ10 F modular stereo-fluorescence microscope (Leica Microsystems, Milton Keynes, UK). Specific band pass filter sets for FITC and Texas red were used to prevent bleed through from one channel to the next. Images were then processed and analysed using Leica Application Suite (Leica Microsystems, Milton Keynes, UK).

### Wide-field fluorescence microscopy

Inoculated coverslips were stained as detailed above at days 1, 3 and 5 with FilmTracer™ LIVE/DEAD^®^ Biofilm Viability Kit to visualise biofilm formation. Images were collected on a Olympus BX51 upright microscope using a 40x/0.75 UPlanFLN objective and captured using a Coolsnap™ EZ camera (Photometrics, Tucson, AZ, USA) through MetaVue™ Software (Molecular Devices, Sunnyvale, CA, USA). Specific band pass filter sets for FITC and Texas red were used to prevent bleed through from one channel to the next. Images were then processed and analysed using ImageJ (http://rsb.info.nih.gov/ij).

Wound tissue explants were embedded in optical cutting temperature (OCT) compound (KP-CryoCompound, Klinipath, Duiven, Netherlands) at days 1, 3 and 5, then snap-frozen in liquid nitrogen and stored at −80 °C before cryosectioning. Ten micrometer sections of biofilm-infected tissue explants were prepared with the use of a cryostat microtome (OTF5000, Bright Instruments Ltd, UK). Each section was fixed in cold acetone for 10 min, which was then allowed to evaporate at room temperature. Slides were immersed in sterilised PBS to remove residual OCT and 50 µl aliquot of Concanavalin-A (Con-A) fluorescent stain was added gently to the section, and then incubated overnight at 4 °C in the dark. The sections were then rinsed gently with PBS to remove excess stain. Sections were counterstained with 4′,6-diamidino-2-phenylindole (DAPI) or SYTO^®^ 9 by incubating for a further 15 min at room temperature in the dark. Sections were finally rinsed gently with PBS and images were collected on a Olympus BX51 upright microscope using a 40x/0.75 UPlanFLN objective and captured using a Coolsnap™ EZ camera (Photometrics, Tucson, AZ, USA) through MetaVue™ Software (Molecular Devices, Sunnyvale, CA, USA). Specific band pass filter sets for DAPI, FITC and Texas red were used to prevent bleed through from one channel to the next. Images were then processed and analysed using ImageJ (http://rsb.info.nih.gov/ij).

### Scanning electron microscopy

Wound tissue explants at day 1, 3, and 5 were fixed in 0.1 M HEPES supplemented with 4% paraformaldehyde and 2.5% glutaraldehyde for 1 h at room temperature and then stored at 4 °C. Each explant was washed 5 times with double distilled water at 5 min intervals and then left in 1% osmium tetroxide for 1 h. Each explant was washed 3 times with double distilled water at 5 min intervals and then dehydrated in a graded ethanol series of 30%, 50%, 70%, 90%, 100% and 100% at 30 min intervals. The explants were then dried in a K850 critical point drier (Quorum Technologies Ltd, Laughton, UK). Subsequently, each explant was mounted on a scanning electron microscopy (SEM) stub and stored in a desiccator until sputter-coated with gold and palladium using an argon gas sputter coating unit. The explants were imaged using SEM (Quanta FEG 250, FEI, Hillsboro, OR, USA) and digital images were captured for qualitative analysis.

### Haematoxylin and Eosin

Wound tissue explants at days 1, 3 and 5 were embedded in OCT compound, snap-frozen in liquid nitrogen and stored at −80 °C. Ten micrometer sections were prepared with the use of a cryostat. Sections were stained for nuclei with haematoxylin (Sigma-Aldrich, Saint Louis, USA) and cytoplasm counterstained with eosin (Sigma-Aldrich, Saint Louis, USA) in a Varistain™ 24-4 Automatic Slide Stainer (ThermoFisher Scientific, Basingstoke, UK). This involved fixation of sections for 2 min in 70% industrial methylated spirits (IMS), followed by a 2 min wash in water. Sections were stained for 2 min in haematoxylin Gills 2, and then in water. Tissue sections were then washed in 5% acetic acid and dehydrated in graded IMS/ethanol (70%, 90% and 100%). Sections were counterstained with alcoholic eosin Y solution for 90 s followed by three 100% ethanol washes. Finally, slides were clarified with xylene and then mounted in mounting media. Images were acquired using a 20x/0.80 Plan Apo objective using the Pannoramic 250 Flash II slide scanner (3D Histech Ltd, Budapest, Hungary). Images were then processed and analysed using CaseViewer 2.0 software (3D Histech Ltd, Budapest, Hungary).

### Explant viability

Explant viability of un-inoculated wound tissue explants at days 1, 3 and 5 were assessed using the XTT cell proliferation assay^27^. The second generation colourless or slightly yellow tetrazolium dye is reduced to a soluble brightly-coloured orange derivative. This is achieved by breaking apart the positively charged quaternary tetrazole ring by a mix of cellular effectors including mitochondrial activity^41^. A saturated solution was prepared from XTT reagent (Sigma-Aldrich, Poole, UK) in 1 × PBS at a concentration of 0.5 g/L. To activate XTT, 100 µl of 1 mM menadione (Sigma-Aldrich, Saint Louis, USA) was added to every 10 ml of XTT solution used. Wound explants were placed into a sterile 24-well Corning® Costar® cell culture plate and 400 µl/well activated XTT solution was added. Following incubation for 2 h at 37 °C in the dark, the colour change was measured using a POLARStar Omega spectrophotometer (BMG Labtech, Ortenberg, Germany) at an absorbance of 490 nm. Results were processed using Omega software (BMG Labtech, Ortenberg, Germany). Triplicate samples for each time point and wound model were prepared and duplicate independent experiments were performed.

### Biofilm metabolic activity

The metabolic activity of biofilms grown on coverslips and wound tissue explants at days 1, 3 and 5 were assessed using the XTT cell proliferation assay with the biofilm separated from the substrate. To separate the biofilms, the substrates were vortexed for 30 s in 1 ml PBS before and after placement in an ultrasonic bath at 60 Hz and 60 W for 5 min. The substrate was removed and the biofilm was allowed to sediment by centrifugation at 12000 × g for 5 min. A saturated solution of XTT was prepared and activated as described above. PBS was removed and 400 µl/well activated XTT solution was added. Samples were vortexed for 30 s to re-suspend the biofilm. Following incubation for 2 h at 37 °C in the dark, the colour change was measured using a POLARStar Omega spectrophotometer (BMG Labtech, Ortenberg, Germany) at an absorbance of 490 and 630 nm. Results were processed using Omega software (BMG Labtech, Ortenberg, Germany). A corrected OD was calculated for inoculated tissue explants by normalising to the metabolic activity of corresponding control (un-inoculated) explants. Triplicate samples for each time point, bacterial strain, wound model and controls were prepared and duplicate independent experiments were performed.

### Biofilm biomass

Biofilms were separated from their respective substrate as described above. Fluorescent nucleic acid Quant-iT PicoGreen dsDNA reagent (Molecular Probes Inc., USA) was used for quantifying dsDNA in solution. DNA was extracted from the biofilms using the QIAamp DNA Mini Kit (QIAGEN, Valencia, CA), according to the manufacturer’s recommendations. The DNA and PicoGreen reagent were mixed thoroughly in the well before fluorometric analysis at 492 nm (BMG Labtech, UK). The lambda DNA within the Quant-iT kit was used to construct the standard curve (concentration range 0–1000 ng/ml) according to the manufacturer’s instructions and measured alongside the samples (100 µl per well; Corning Costar, UK). Triplicate samples for each time point, bacterial strain, wound model and controls (un-inoculated) were prepared and duplicate independent experiments were performed. DNA extraction of control (un-inoculated) samples were undertaken to quantify the amount of dsDNA present from the cutaneous explant substrates as a result of the biofilm separation technique used.

### Gas chromatography-mass spectrometry

Gas chromatography-mass spectrometric (GCMS) analyses were performed using an Agilent 5977A MSD mass spectrometer (Agilent Technologies, Santa Clara, CA, USA) linked to GC (Agilent 7890B GC system, Agilent Technologies, Santa Clara, CA, USA) which was serviced by an autosampler (PAL RSI 85 autosampler system, Agilent Technologies, Santa Clara, CA, USA). Coverslips and wound tissue explants sealed in headspace vials at days 0, 1, 3 and 5 were agitated at 37 °C for 5 min before a 1 ml headspace gas sample was collected using an Agilent gas-tight syringe (Agilent Technologies, Santa Clara, CA, USA) attached to the autosampler. This was injected directly onto a DB-Wax column (30 m, 0.32 mm inner diameter, 0.25 µm film thickness (Agilent Technologies, Santa Clara, CA, USA)) through an injector (split-less) and separated using 1.02 ml/min column flow. The oven temperature profile was 40 °C for 5 min, increased to 230 °C (10 °C/min) over 19 min and finished with 5 min at 230 °C. VOCs were detected in the mass to charge (m/z) range of 25–350 Da.

Processing of chromatograms was performed by MassHunter qualitative analysis software (Agilent Technologies, Santa Clara, CA, USA) prior to further statistical analyses. Chromatograms were processed by introducing a 2 min solvent delay, noise removal and by comparing each inoculated sample to its corresponding time point control. Peaks based on retention times unique to inoculated samples underwent baseline subtraction and the resulting mass spectra were chemically identified based on forward and reverse match searches of the National Institute of Standards and Technology library. A score of 1000 was rated a perfect match, 900–999 an excellent match, 800–900 a good match and 700–800 a fair match. Proposed chemical compounds were sorted based on forward match and the compound with the highest score. Where the highest probable compound for a peak had already been selected for a preceding peak the next previously unselected compound was selected as the corresponding VOC. Elution order and relative retention time were also taken into account; where it was not possible for the identified compound to have eluted at the determined retention time, one of the lower probability compounds was chosen. The area of each unique chromatographic peak was calculated to provide relative abundances. A peak common to all infected and non-infected samples and controls irrespective of model used or time point assessed, was identified and the area of each unique chromatographic peak was normalised to this common peak to account for GCMS variations over the sampling duration. The relative abundance of this common peak was log transformed in all infected samples across all models and time points (n = 216; mean = 10.7; standard error of the mean = 0.03). A value of 1 was added to all normalised peak areas and subsequently log transformed prior to statistical analyses.

### Statistical analysis

Continuous data were summarised as the mean ± standard error of the mean. Presence or absence of peaks between time points, between bacterial samples and controls were analysed using a one sample t-test. Differences in XTT, PicoGreen and relative abundance of peaks between and within bacterial groups; and between wound model types were analysed using a one way-analysis of variance with accompanying Tukey post hoc analyses. Spearman’s correlation co-efficient was calculated to correlate XTT or PicoGreen to relative abundance of peaks. A P value of < 0.05 was considered statistically significant. Statistical analyses were performed using SPSS for Windows version 22.0 (SPSS, IBM, Armonk, NY, USA) and graphical representation was performed using GraphPad Prism 7 (GraphPad Software, La Jolla, CA, USA).

### Ethics statement

Written informed consent was obtained from patients for tissue samples. Permission was granted by National Health Service Research Ethics committee North West (16/NW/0736). Tissue collection methodology, use of tissue samples in experiments, storage and disposal of tissue samples were performed in accordance with the relevant guidelines and regulations approved by National Health Service Research Ethics committee North West (16/NW/0736).

## Electronic supplementary material

Supplementary Information
