# Supplementary material for: Validation of biofilm formation on human skin wound models and demonstration of clinically translatable bacteria-specific volatile signatures
Source: Sci Rep. 2018 Jun 21;8:9431. doi: 10.1038/s41598-018-27504-z (PMC6013498; doi:10.1038/s41598-018-27504-z)
Supplement: Supplementary file 1 — Supplementary Information [file 41598_2018_27504_MOESM1_ESM.pdf]

Supplementary Information

Validation of biofilm formation on human skin wound models and demonstration of clinically translatable bacteria-specific volatile signatures

Mohammed Ashrafi, Lilyann Novak-Frazer, Matthew Bates, Mohamed Baguneid, Teresa Alonso-Rasgado, Guoqing Xia, Riina Rautemaa-Richardson, Ardeshir Bayat*

*SI correspondence to: ardeshir.bayat@manchester.ac.uk

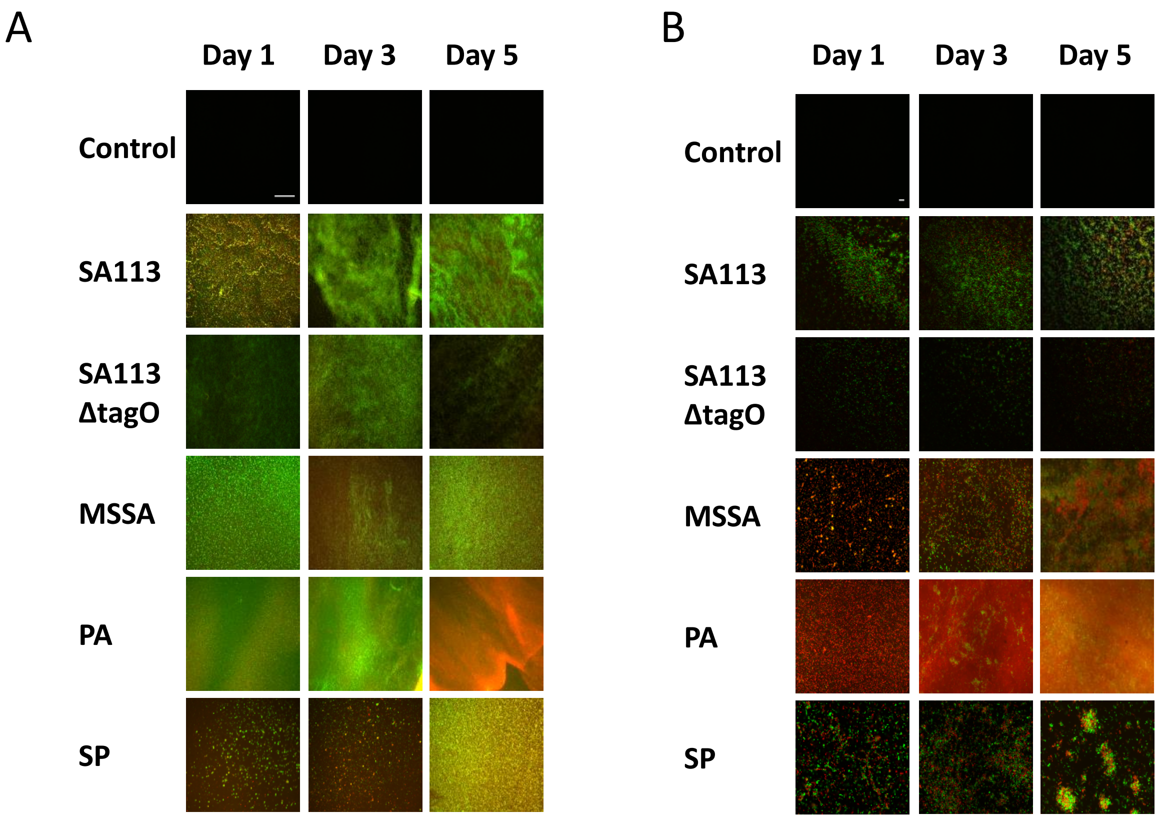

Figure S1. ***In vitro* model of biofilm formation**. Aerial view of biofilms on coverslips showing viable bacteria stained green with the nucleic acid stain SYTO® 9 and dead cells and extracellular DNA stained red with the nucleic acid stain propidium iodide (PI). (**A)** Stereo-fluorescence microscopy. Scale bar: 250µm. (**B)** Wide-field fluorescence microscopy. Scale bar: 10µm is equal in all images.

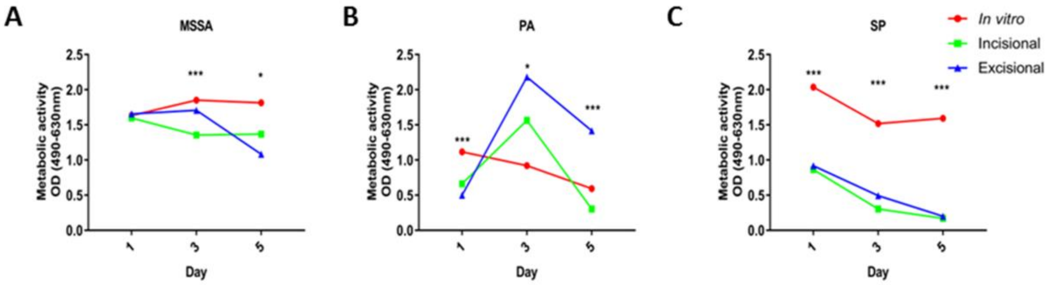

Figure S2. **Inter-model comparisons of metabolic activity of biofilms**. Inter-model comparisons of metabolic activity of MSSA (**A**), PA (**B**) and SP (**C**) biofilms following XTT reduction assay. Mean ± standard error of the mean (n=6), * P < 0.05, ** P < 0.01, *** P < 0.001, as determined by one-way analysis of variances with accompanying Tukey post hoc analyses.

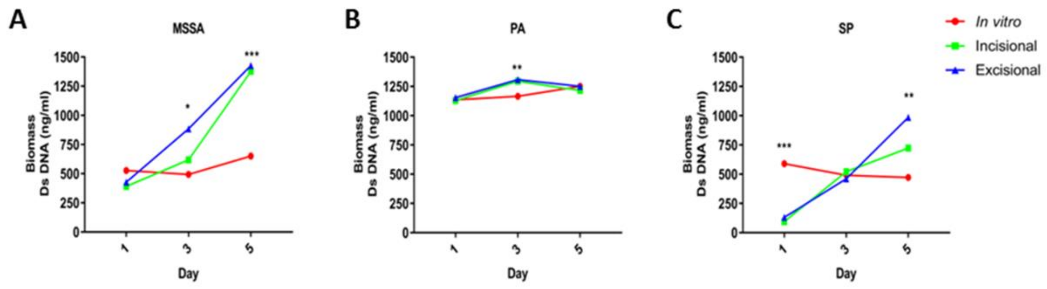

Figure S3. **Inter-model comparisons of biomass of biofilms**. Inter-model comparisons of biomass of MSSA (**A**), PA (**B**) and SP (**C**) biofilms following Quant-iT PicoGreen dsDNA reagent assay. Mean ± standard error of the mean (n=6), * P < 0.05, ** P < 0.01, *** P < 0.001, as determined by one-way analysis of variances with accompanying Tukey post hoc analyses.

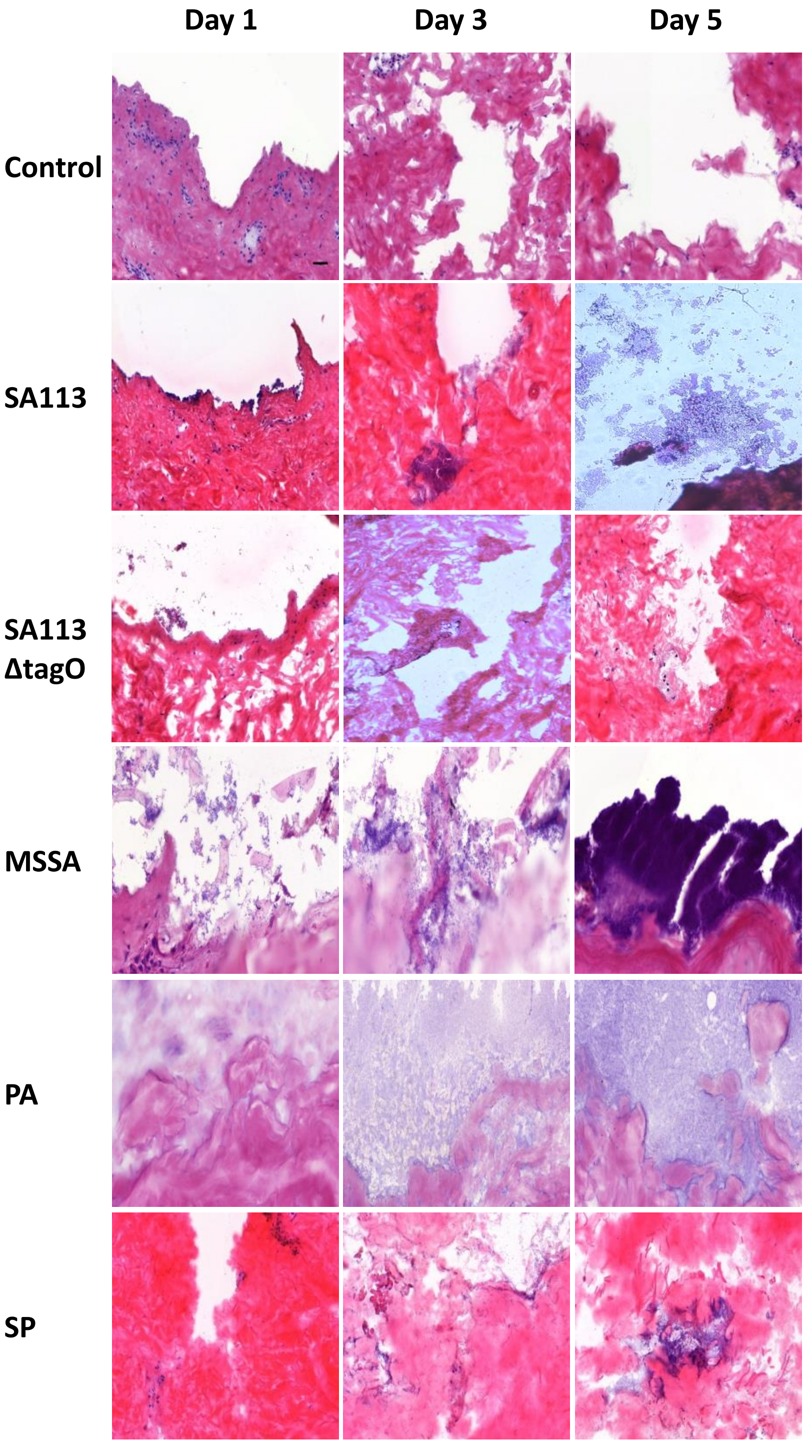

Figure S4. **Haematoxylin and eosin stained microscopy of biofilms of *ex vivo* incisional wound model.** Wound tissue is stained pink and biofilm material blue or purple. Scale bar: 20 µm is equal in all images.

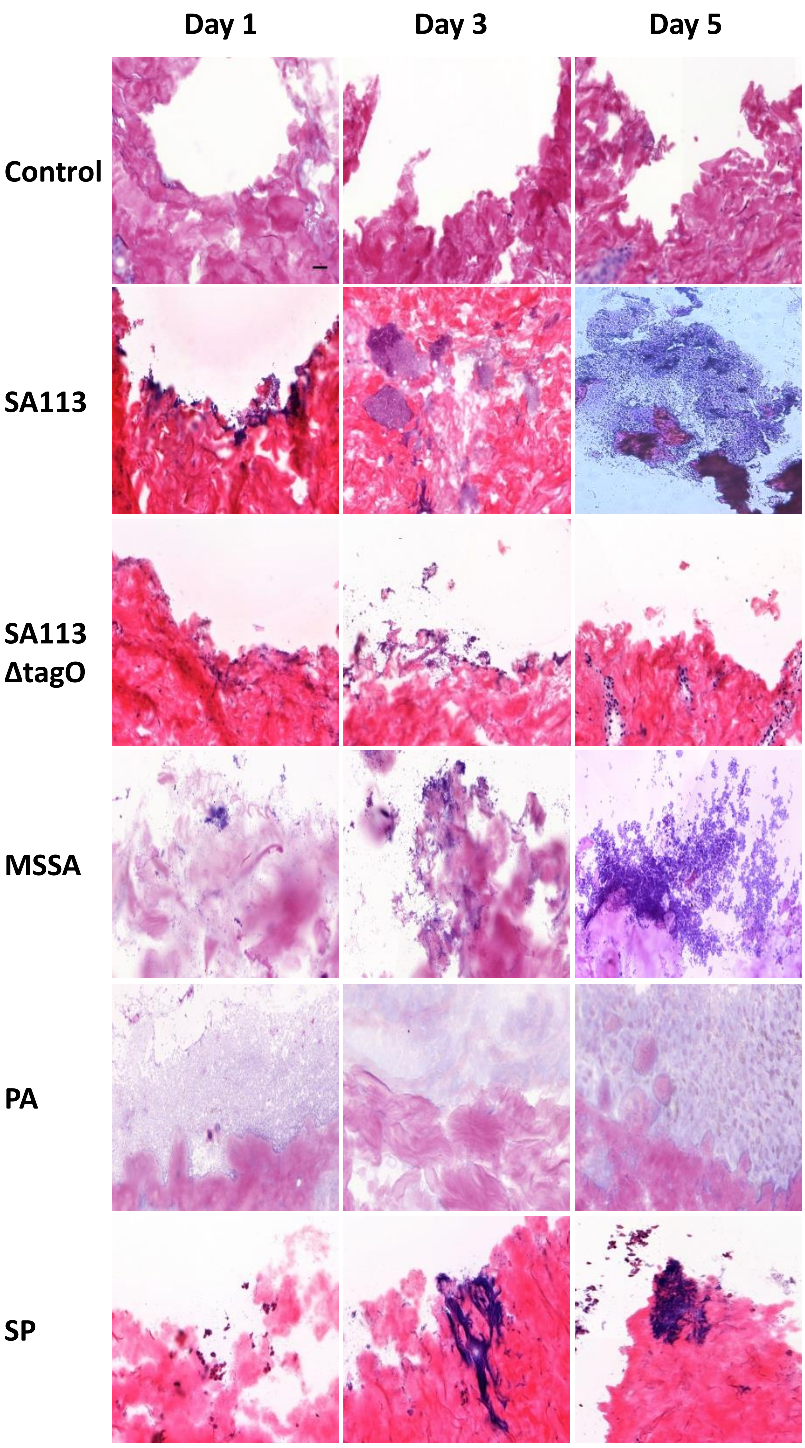

Figure S5. Haematoxylin and eosin stained microscopy of biofilms of *ex vivo* excisional wound model. Wound tissue is stained pink and biofilm material blue or purple. Scale bar: 20 µm is equal in all images.

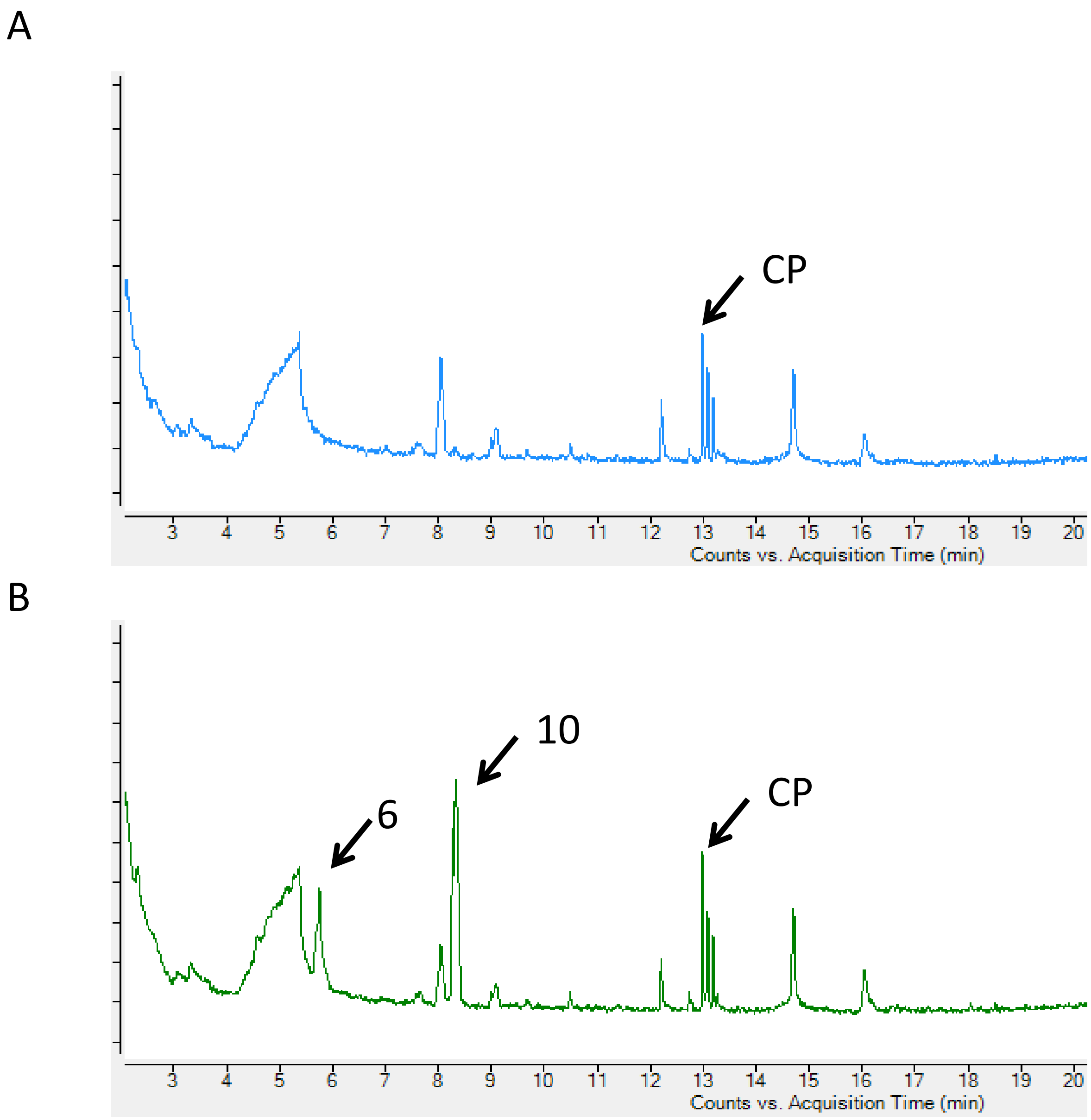

Figure S6. Representative chromatograms at day 1 of (A) *ex vivo* excisional cutaneous wound explant control and (B) *ex vivo* excisional cutaneous wound explant inoculated with MSSA. 6 - 2-methyl-1-propanol; 10 - 3-methyl-1-butanol; CP – common peak. Snapshots of chromatograms up to 20 min after which there were no further peaks. Non-labelled peaks were shared between inoculated and control samples.

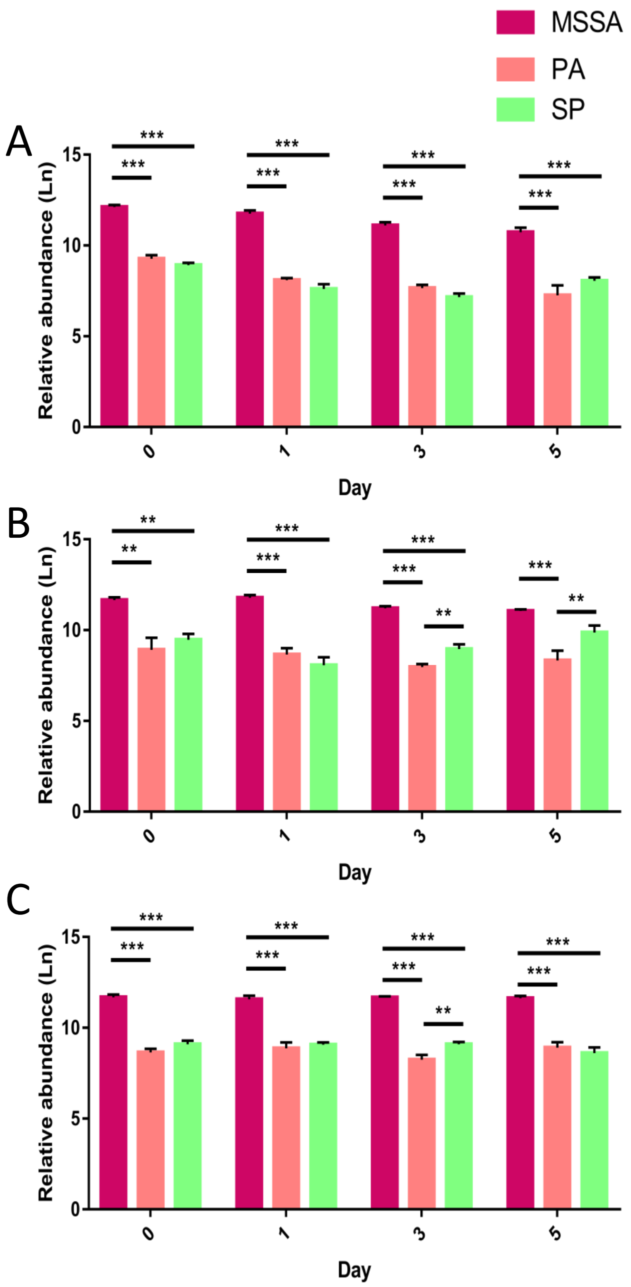

Figure S7. **The presence of 2-methyl-1-propanol among bacterial species and in different wound models. (A)** *in vitro*; (**B)** *ex vivo* incisional and (**C)** *ex vivo* excisional wound model. Relative abundance of 2-methyl-1-propanol on days 0, 1, 3 and 5 produced by MSSA (pink), PA (peach) and SP (green) biofilms. Mean ± standard error of the mean (n=6), * P < 0.05, ** P < 0.01, *** P < 0.001, as determined by one-way analysis of variances with accompanying Tukey post hoc analyses.

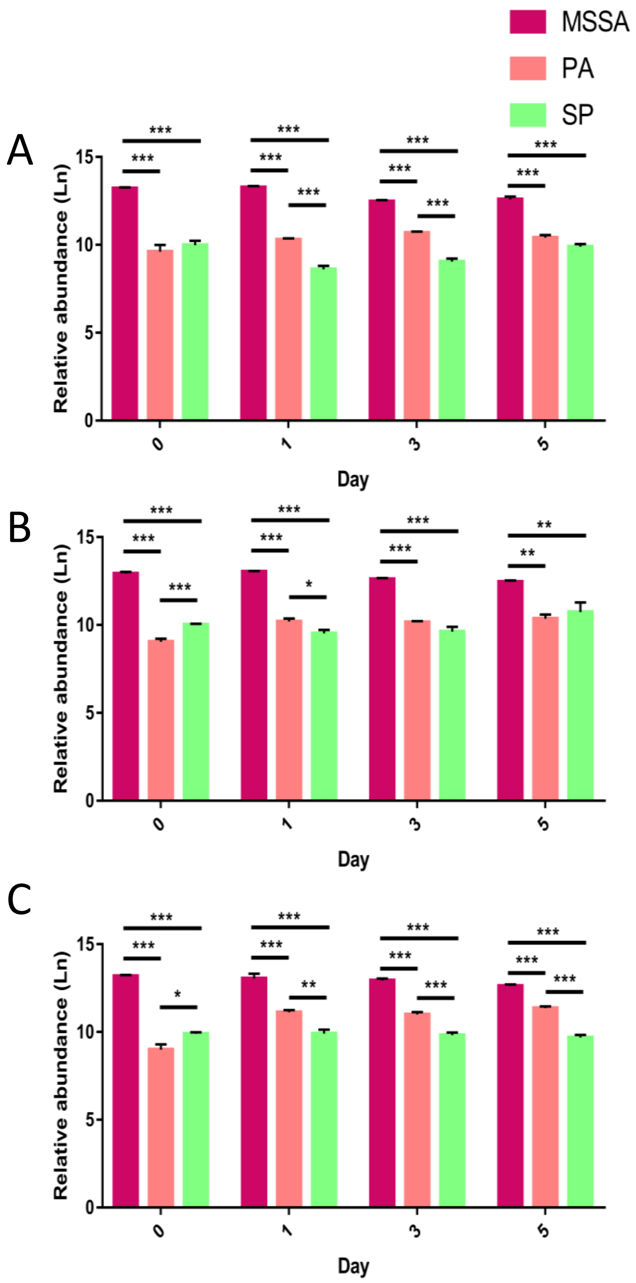

Figure S8. **The presence of 3-methyl-1-butanol among bacterial species and in different wound models. (A)** *in vitro*; (**B)** *ex vivo* incisional; and (**C)** *ex vivo* excisional wound model. Relative abundance of 3-methyl-1-butanol on days 0, 1, 3 and 5 produced by MSSA (pink), PA (peach) and SP (green) biofilms. Mean ± standard error of the mean (n=6). * $P < 0.05$, ** $P < 0.01$, *** $P < 0.001$, as determined by one-way analysis of variances with accompanying Tukey post hoc analyses.

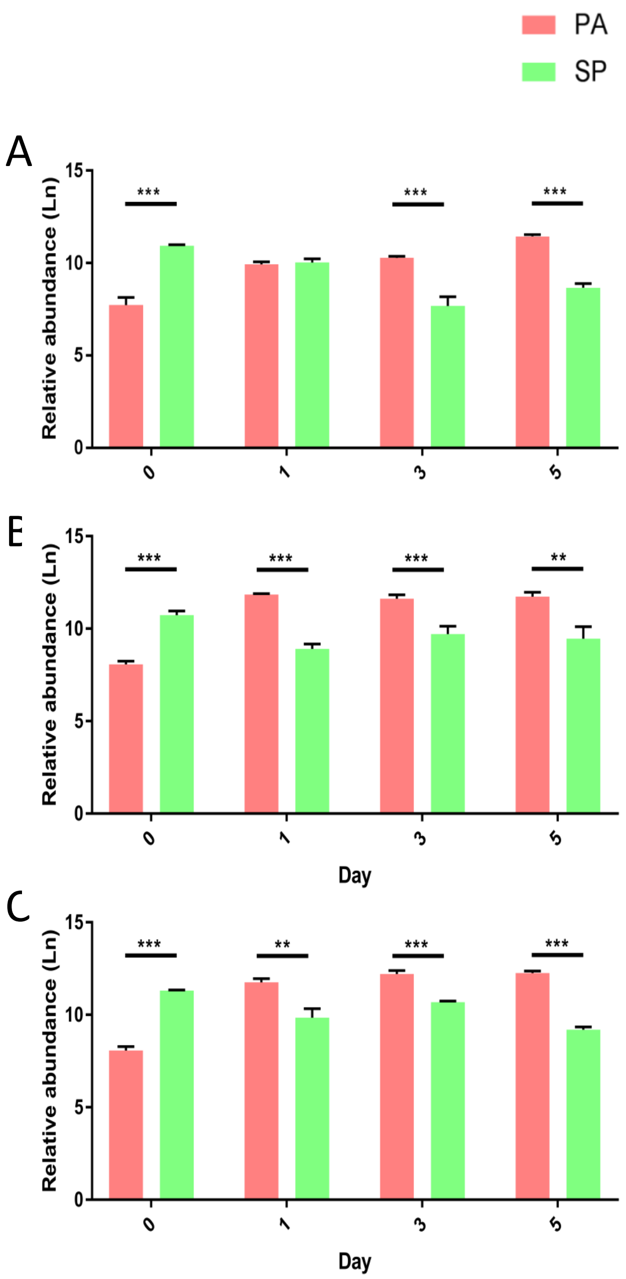

Figure S9. **The presence of 1-undecene among bacterial species and in different wound models (except MSSA). (A)** *in vitro*; (**B)** *ex vivo* incisional; and (**C)** *ex vivo* excisional wound model. Relative abundance of 1-undecene on days 0, 1, 3 and 5 produced by PA (peach) and SP (green) biofilms. Mean ± standard error of the mean (n=6), * $P < 0.05$, ** $P < 0.01$, *** $P < 0.001$, as determined by one-way analysis of variances with accompanying Tukey post hoc analyses.

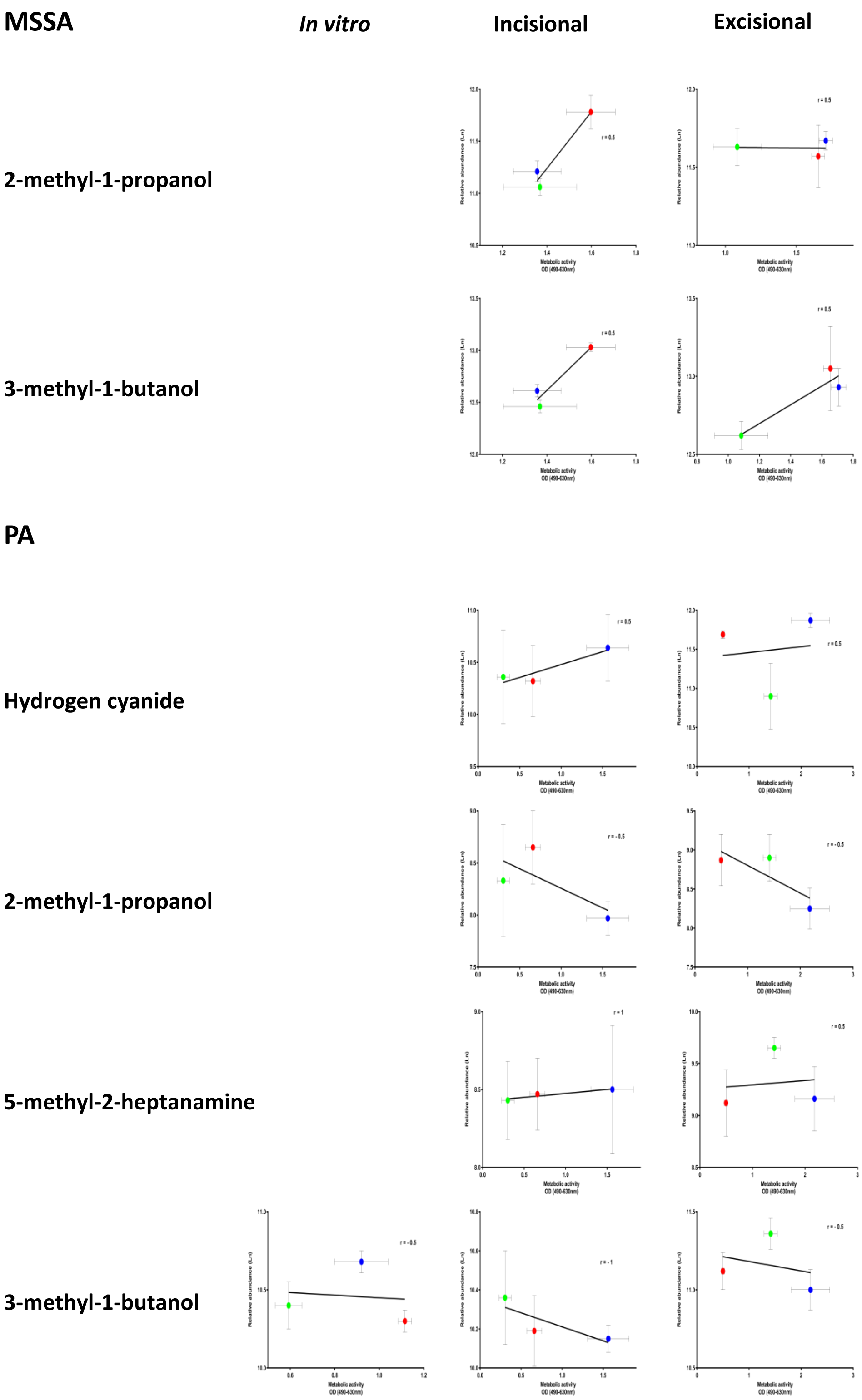

Figure S10. Correlations between biofilm metabolic activity and abundance of VOCs across models on day 1 (red), 3 (blue) and 5 (green). Mean ± SEM (n=6).

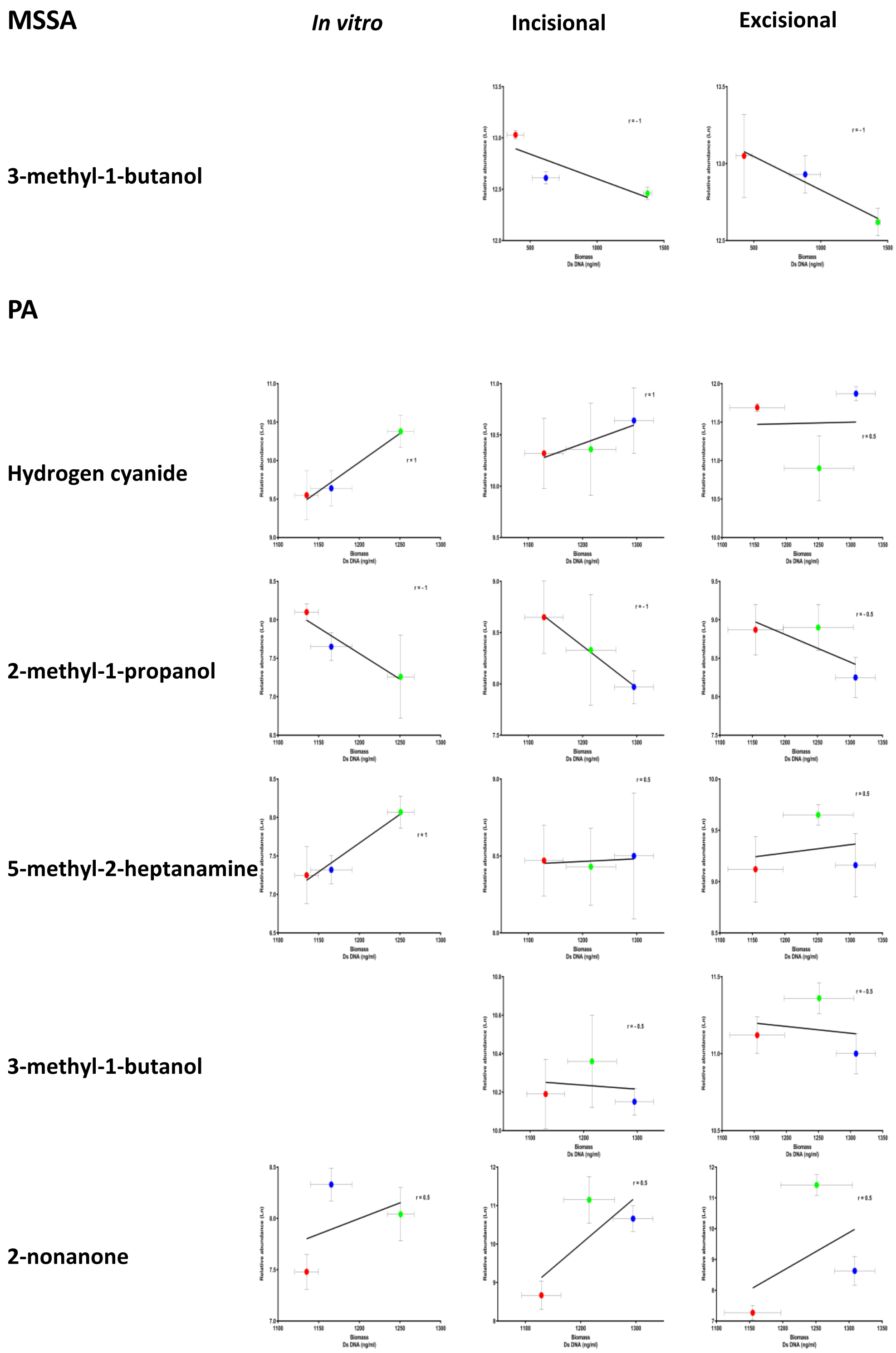

Figure S11. Correlations between biofilm biomass and abundance of VOCs across models on day 1 (red), 3 (blue) and 5 (green). Mean ± SEM (n=6).

Table S1. Proposed chemical compound of the peaks identified by GC-MS based on National Institute of Standards and Technology library spectral matching (spectra of all three species combined).

Peak	Proposed compound	Match	R. Match	RI	Prob (%)
1	3-methylbutanal	818	870	652	69.0
2	ethanol	831	901	427	93.3
3	pentanal	858	881	699	35.2
4	2-butanol	671	724	598	8.3
5	hydrogen cyanide	857	961	300	99.0
6	2-methyl-1-propanol	819	852	625	88.5
7	5-methyl-2-hexanamine	657	827		44.1
8	5-methyl-2-heptanamine	669	715		7.28
9	1-undecene	873	874	1091	7.8
10	3-methyl-1-butanol	912	918	736	67.0
11	2-nonanone	915	917	1092	76.4
12	2-undecanone	914	922	1294	80.7

R. Match – reverse match; RI – retention index; Prob – probability

Table S2. VOCs identified from biofilms of different bacterial species in the *in vitro* model indicating the statistical differences between time point measurements

VOC	D0 vs. D1	D0 vs. D3	D0 vs. D5	D1 vs. D3	D1 vs. D5	D3 vs. D5
**MSSA**						
**3-methylbutanal**	> 0.05	**< 0.001**	**< 0.001**	**< 0.001**	**< 0.001**	> 0.05
**pentanal**	**0.001**	**< 0.001**	**< 0.001**	**< 0.001**	**<0.001**	> 0.05
**2-methyl-1-propanol**	> 0.05	**0.006**	**< 0.001**	> 0.05	**0.005**	> 0.05
**3-methyl-1-butanol**	> 0.05	**< 0.001**	**0.002**	**< 0.001**	**0.001**	> 0.05
**PA**						
**hydrogen cyanide**	**< 0.001**	**< 0.001**	**< 0.001**	> 0.05	> 0.05	> 0.05
**2-methyl-1-propanol**	> 0.05	**0.007**	**0.001**	> 0.05	> 0.05	> 0.05
**5-methyl-2-hexanamine**	**< 0.001**	**< 0.001**	**< 0.001**	**0.004**	**< 0.001**	**< 0.001**
**5-methyl-2-heptanamine**	**< 0.001**	**< 0.001**	**< 0.001**	> 0.05	> 0.05	> 0.05
**1-undecene**	**< 0.001**	**< 0.001**	**< 0.001**	> 0.05	**0.001**	**0.009**
**3-methyl-1-butanol**	> 0.05	**0.01**	> 0.05	> 0.05	> 0.05	> 0.05
**2-nonanone**	**< 0.001**	**< 0.001**	**< 0.001**	**0.012**	> 0.05	> 0.05
**SP**						
**ethanol**	**< 0.001**	**< 0.001**	**< 0.001**	**< 0.001**	**< 0.001**	> 0.05
**2-butanol**	**< 0.001**	**< 0.001**	**< 0.001**	**< 0.001**	**< 0.001**	> 0.05
**2-methyl-1-propanol**	**0.001**	**< 0.001**	**0.03**	> 0.05	> 0.05	**0.022**
**1-undecene**	> 0.05	**< 0.001**	**< 0.001**	**< 0.001**	**0.016**	> 0.05
**3-methyl-1-butanol**	**< 0.001**	**0.013**	> 0.05	> 0.05	**0.001**	**0.025**

P values (one-way analysis of variances with accompanying Tukey post hoc analyses); D0 – planktonic phase; D 1, 3 and 5 – biofilm phase (days)

Table S3. VOCs identified from biofilms of different bacterial species in the *ex vivo* human cutaneous incisional model indicating the statistical differences between time points

VOC	D0 vs. D1	D0 vs. D3	D0 vs. D5	D1 vs. D3	D1 vs. D5	D3 vs. D5
**MSSA**						
**3-methylbutanal**	**< 0.001**	**< 0.001**	**< 0.001**	> 0.05	> 0.05	> 0.05
**pentanal**	**< 0.001**	**< 0.001**	**< 0.001**	> 0.05	> 0.05	> 0.05
**2-methyl-1-propanol**	> 0.05	> 0.05	**0.015**	**0.021**	**0.003**	> 0.05
**3-methyl-1-butanol**	> 0.05	**0.019**	**0.001**	**0.001**	**< 0.001**	> 0.05
**PA**						
**hydrogen cyanide**	**< 0.001**	**< 0.001**	**< 0.001**	> 0.05	> 0.05	> 0.05
**2-methyl-1-propanol**	> 0.05	> 0.05	> 0.05	> 0.05	> 0.05	> 0.05
**5-methyl-2-hexanamine**	**< 0.001**	**< 0.001**	**< 0.001**	> 0.05	> 0.05	> 0.05
**5-methyl-2-heptanamine**	**< 0.001**	**< 0.001**	**< 0.001**	> 0.05	> 0.05	> 0.05
**1-undecene**	**< 0.001**	**< 0.001**	**< 0.001**	> 0.05	> 0.05	> 0.05
**3-methyl-1-butanol**	**0.001**	**0.001**	**< 0.001**	> 0.05	> 0.05	> 0.05
**2-nonanone**	**< 0.001**	**< 0.001**	**< 0.001**	**0.009**	**0.001**	> 0.05
**2-undecanone**	**< 0.001**	**< 0.001**	**< 0.001**	> 0.05	> 0.05	> 0.05
**SP**						
**ethanol**	**< 0.001**	**< 0.001**	**< 0.001**	> 0.05	> 0.05	> 0.05
**2-butanol**	**< 0.001**	**< 0.001**	**< 0.001**	> 0.05	> 0.05	> 0.05
**2-methyl-1-propanol**	> 0.05	> 0.05	> 0.05	> 0.05	**0.01**	> 0.05
**1-undecene**	**0.03**	> 0.05	> 0.05	> 0.05	> 0.05	> 0.05
**3-methyl-1-butanol**	> 0.05	> 0.05	> 0.05	> 0.05	> 0.05	> 0.05

P values (one-way analysis of variances with accompanying Tukey post hoc analyses); D0 – planktonic phase; D 1, 3 and 5 – biofilm phase (days)

Table S4. VOCs identified from biofilms of different bacterial species in the *ex vivo* human cutaneous excisional model indicating the statistical differences between time points

VOC	D0 vs. D1	D0 vs. D3	D0 vs. D5	D1 vs. D3	D1 vs. D5	D3 vs. D5
**MSSA**						
**3-methylbutanal**	**< 0.001**	**< 0.001**	**< 0.001**	> 0.05	> 0.05	> 0.05
**pentanal**	**< 0.001**	**< 0.001**	**< 0.001**	> 0.05	> 0.05	> 0.05
**2-methyl-1-propanol**	> 0.05	> 0.05	> 0.05	> 0.05	> 0.05	> 0.05
**3-methyl-1-butanol**	> 0.05	> 0.05	> 0.05	> 0.05	> 0.05	> 0.05
**PA**						
**hydrogen cyanide**	**< 0.001**	**< 0.001**	**< 0.001**	> 0.05	> 0.05	**0.024**
**2-methyl-1-propanol**	> 0.05	> 0.05	> 0.05	> 0.05	> 0.05	> 0.05
**5-methyl-2-hexanamine**	**< 0.001**	**< 0.001**	**< 0.001**	**0.017**	**0.039**	> 0.05
**5-methyl-2-heptanamine**	**< 0.001**	**< 0.001**	**< 0.001**	> 0.05	> 0.05	> 0.05
**1-undecene**	**< 0.001**	**< 0.001**	**< 0.001**	> 0.05	> 0.05	> 0.05
**3-methyl-1-butanol**	**< 0.001**	**< 0.001**	**< 0.001**	> 0.05	> 0.05	> 0.05
**2-nonanone**	**< 0.001**	**< 0.001**	**< 0.001**	**0.028**	**< 0.001**	**< 0.001**
**2-undecanone**	**< 0.001**	**< 0.001**	**< 0.001**	> 0.05	> 0.05	> 0.05
**SP**						
**ethanol**	**0.001**	> 0.05	**< 0.001**	**< 0.001**	**< 0.001**	**< 0.001**
**2-butanol**	**< 0.001**	**0.011**	**< 0.001**	**< 0.001**	**< 0.001**	**< 0.001**
**2-methyl-1-propanol**	> 0.05	> 0.05	> 0.05	> 0.05	> 0.05	> 0.05
**1-undecene**	**0.004**	> 0.05	**< 0.001**	> 0.05	> 0.05	**0.003**
**3-methyl-1-butanol**	> 0.05	> 0.05	> 0.05	> 0.05	> 0.05	> 0.05

P values (one-way analysis of variances with accompanying Tukey post hoc analyses); D0 – planktonic phase; D 1, 3 and 5 – biofilm phase (days)
